# A social marketing approach to implementing evidence-based practice in VHA QUERI: the TIDES depression collaborative care model

**DOI:** 10.1186/1748-5908-4-64

**Published:** 2009-09-28

**Authors:** Jeff Luck, Fred Hagigi, Louise E Parker, Elizabeth M Yano, Lisa V Rubenstein, JoAnn E Kirchner

**Affiliations:** 1 VA Greater Los Angeles HSR&D Center of Excellence, VA Greater Los Angeles Healthcare System, Sepulveda, CA, USA; 2 Department of Health Services, UCLA School of Public Health, Los Angeles, CA, USA; 3 Department of Medicine, UCLA David Geffen School of Medicine, Los Angeles, CA, USA; 4 Center for Mental Healthcare and Outcomes Research, North Little Rock, AR, USA; 5 South Central Mental Illness Research Education and Clinical Center (MIRECC), Little Rock, AR, USA; 6 University of Arkansas Medical Sciences (UAMS) Center, Little Rock, AR, USA

## Abstract

**Abstract:**

Collaborative care models for depression in primary care are effective and cost-effective, but difficult to spread to new sites. Translating Initiatives for Depression into Effective Solutions (TIDES) is an initiative to promote evidence-based collaborative care in the U.S. Veterans Health Administration (VHA). Social marketing applies marketing techniques to promote positive behavior change. Described in this paper, TIDES used a social marketing approach to foster national spread of collaborative care models.

**TIDES social marketing approach:**

The approach relied on a sequential model of behavior change and explicit attention to audience segmentation. Segments included VHA national leadership, Veterans Integrated Service Network (VISN) regional leadership, facility managers, frontline providers, and veterans. TIDES communications, materials and messages targeted each segment, guided by an overall marketing plan.

**Results:**

Depression collaborative care based on the TIDES model was adopted by VHA as part of the new Primary Care Mental Health Initiative and associated policies. It is currently in use in more than 50 primary care practices across the United States, and continues to spread, suggesting success for its social marketing-based dissemination strategy.

**Discussion and conclusion:**

Development, execution and evaluation of the TIDES marketing effort shows that social marketing is a promising approach for promoting implementation of evidence-based interventions in integrated healthcare systems.

## Background

Implementing evidence-based interventions in a healthcare provider organization is a challenging endeavor, requiring changes in attitudes, beliefs and behavior [1]. Mandating change may be a seemingly simple course of action, but is rarely effective [2-4], especially because clinicians have a strong occupational culture and enjoy a high degree of professional autonomy in healthcare organizations [5]. Rather, change is most likely to occur when organizational members' attitudes and beliefs are concordant with the desired change, and they are willing to behave accordingly.

These challenges are amplified in an integrated healthcare organization with multiple points of care (sites) that exhibit significant variation in local cultures and circumstances. For example, USA's Veterans Health Administration (VHA) is a nationwide system of outpatient facilities and medical centers, which is organized into 21 regional Veterans Integrated Service Networks (VISNs) and supervised by a national Central Office [6]. Therefore, successful national implementation in VHA depends upon decisions made at local, regional, and national levels. Other large, complex, integrated healthcare systems, such as the British National Health Service and USA staff model health maintenance organizations, face similar challenges.

Marketing is a discipline whose core goal is affecting behavior. Specifically, commercial marketing campaigns aim to influence consumers' purchasing decisions [7], whereas *social *marketing campaigns promote socially desirable behaviors such as exercise, recycling, or smoking cessation [8,9]. Although most healthcare social marketing efforts have been aimed at consumers, recently there has been interest in utilizing these techniques to effect change among healthcare providers [10,11]. If a social marketing approach can help to address the challenges of decision-making and behavior change in healthcare provider organizations, it may facilitate national implementation of evidence-based interventions.

### QUERI and collaborative depression care in VHA

This article follows on from a *Series *of articles documenting implementation science frameworks and tools developed by USA's Department of Veterans Affairs (VA) Quality Enhancement Research Initiative (QUERI). QUERI is briefly outlined in Table 1 and described in more detail in previous publications [12,13]. The *Series' *introductory article [14] highlights aspects of QUERI related specifically to implementation science and describes additional types of articles contained in the *QUERI Series*.

**Table 1:** The VA Quality Enhancement Research Initiative (QUERI).

The U.S. Department of Veterans Affairs' (VA) Quality Enhancement Research Initiative (QUERI) was launched in 1998. QUERI was designed to harness VA's health services research expertise and resources in an ongoing system-wide effort to improve the performance of the VA healthcare system and, thus, quality of care of veterans. QUERI researchers collaborate with VA policy and practice leaders, clinicians, and operations staff to implement appropriate evidence-based practices into routine clinical care. They work within distinct disease- or condition-specific QUERI Centers and utilize a standard six-step process:
1) Identify high-risk/high-volume diseases or problems,
2) Identify best practices,
3) Define existing practice patterns and outcomes across the VA and current variation from best practices,
4) Identify and implement interventions to promote best practices,
5) Document that best practices improve outcomes, and
6) Document that outcomes are associated with improved health-related quality of life.
Within Step 4, QUERI implementation efforts generally follow a sequence of four phases to enable the refinement and spread of effective and sustainable implementation programs across multiple VA medical centers and clinics. The phases include:
1) Single-site pilot,
2) Small-scale, multi-site implementation trial,
3) Large-scale, multi-region implementation trial, and
4) System-wide rollout
Researchers employ additional QUERI frameworks and tools, as highlighted in this *Series*, to enhance achievement of each project's quality improvement and implementation science goals.

**Figure 1:**
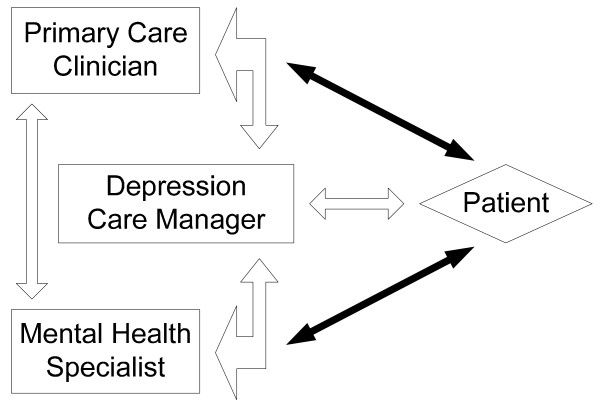
**Translating Initiatives for Depression into Effective Solutions (TIDES) model of collaborative care for depression**. **Source**: TIDES Fact Sheet.

The Mental Health QUERI (MH-QUERI) leads the evaluation and dissemination of Translating Initiatives for Depression into Effective Solutions (TIDES), an initiative for developing and spreading collaborative care models for depression within VHA primary care practices [15]. In collaborative care models for depression, shown in Figure 1, a care manager assists primary care providers with managing depressed patients and facilitates collaboration between primary care and mental health.

Carefully designed studies have generated clear evidence of the effectiveness [16] and cost-effectiveness [17,18] of collaborative care for depression in non-VHA and VHA [19,20] settings. The TIDES model was first implemented in seven "first-generation" VHA sites in three VISNs during 2002-2004. From 2004 through 2008, implementation was expanded to additional second-generation sites in those VISNs, as well as in one additional VISN. Evaluators concurrently collected data at control sites, and also collected data for cost-effectiveness analyses. The collaborative care model developed by TIDES, in partnership with first- and second-generation sites, was incorporated into a national funding initiative for primary care mental health in 2006, and incorporated into national policy in 2008.

Therefore, TIDES can be described as being in Phase 3 of QUERI Step 4 (see Table 1). Formative evaluation activities [21], such as a systematic program of stakeholder interviews and measurement of program penetration and utilization, and spread to additional sites are ongoing.

MH-QUERI works actively with VHA leaders and stakeholders to implement the TIDES collaborative depression care model nationally [22]. Thus it developed a TIDES National Dissemination (Spread) Plan that establishes goals in four areas: 1) Guidelines and Quality Indicators, 2) Training in Clinical Processes and Evidence-Based Quality Improvement, 3) Marketing, and 4) Informatics and Logistics Support. This paper describes how a social marketing approach informed the marketing activities performed under that plan, as well as the underlying marketing theory and associated evaluation activities.

## Social marketing: Applying marketing techniques to social problems

Marketing is "the process of planning and executing the Conception, Pricing, Promotion, and Distribution of ideas, goods, and services to create exchanges that satisfy individual and organizational objectives" [23]. Effective marketing is crucial to the success of for-profit firms. Kotler [9] proposed applying marketing principles to meeting societal objectives, such as improving the health and welfare of individuals and society, rather than corporate ones. He termed this application "social marketing." Social marketing is most commonly used to convince the public to adopt socially beneficial behaviors (e.g., recycling, exercise) and eliminate socially undesirable ones (e.g., littering, overeating).

Andreasen suggests several benchmarks against which to assess a social marketing approach [24]. As described in detail in Table 2, they include: a central emphasis on behavior change as the goal of social marketing efforts, systematic use of audience research, segmentation of target audiences, and use of the traditional "4 P's" of marketing (Product, Price, Place, and Promotion). In the context of implementing a clinical intervention in VHA, the Product would be the benefits resulting from successful implementation; Price is the financial and time cost to the implementing site and its clinicians and staff; Place comprises the computerized or interpersonal means by which the intervention can be provided to individual patients; and Promotion includes the range of techniques used to communicate with target audiences.

**Table 2:** Benchmarks for a social marketing approach.

1. Behavior-change is the benchmark used to design and evaluate interventions.
2. Projects consistently use audience research to: a) understand target audiences at the outset of interventions (i.e., formative research), b) routinely pretest intervention elements before they are implemented, and c) monitor interventions as they are rolled out.

3. There is careful segmentation of target audiences to ensure maximum efficiency and effectiveness in the use of scarce resources.

4. The central element of any influence strategy is creating attractive and motivational exchanges with target audiences.

5. The strategy attempts to use all four Ps of the traditional marketing mix; for example, it is not just advertising or communications. That is, it creates attractive benefit packages (products) while minimizing costs (price) wherever possible, making the exchange convenient and easy (place), and communicating powerful messages through media relevant to--and preferred by--target audiences (promotion).

6. Careful attention is paid to the competition faced by the desired behavior.

**Source**: Andreasen, AR., "**Marketing Social Marketing in the Social Change Marketplace**," *Journal of Public Policy & Marketing*, **21**(1):3-13, 2002, p. 7.

Andreasen further observes that social marketing has important similarities to, and shares techniques with, other behavior change approaches [24]. These approaches include: social learning theory, which emphasizes fostering audience members' efficacy in adopting the new behavior [25]; behavioral reinforcement theory that focuses on providing incentives for behavior change [26]; and health promotion campaigns, often based on models such as PRECEDE-PROCEED [27]. Grol, Wensing, and Eccles recognize the marketing approach as one of several (e.g., educational, external influence, and social interaction approaches) used to implement clinical interventions [28]. Linden and Roberts note that social marketing is a comprehensive model--operating at the community, interpersonal, and individual levels--that shares goals and techniques with other healthcare behavioral change models [29]. The social marketing approach also is consistent with the empirically-based conceptual model for diffusion and dissemination of innovations in health services organizations recently proposed by Greenhalgh and colleagues [30].

Most published evaluations of health-related social marketing report on individual interventions or limited numbers of case studies [31,32]. The Institute for Social Marketing has recently conducted a series of rigorous reviews of the available evidence regarding the effectiveness of social marketing for improving health [33]. They reviewed published reports of 88 interventions targeting diet, exercise, and substance abuse (including smoking). Interventions were included only if they met all of Andreasen's six benchmarks for social marketing (Table 2). Although results could not be aggregated statistically due to the diversity of interventions, the authors conclude that social marketing can be effective, particularly to encourage diet improvement and treatment for substance abuse.

### Social marketing and the promotion of behavior change

Social marketing explicitly recognizes that behavior change is a sequential process. New information and the resulting thoughts prompt the adopter to replace existing behaviors (e.g., exercise or eating habits) with new ones. These new behaviors will become routine only if they are reinforcing (i.e., yield positive outcomes for the adopter). For successful implementation of an evidence-based clinical intervention in VHA, behavior change must occur among three broadly defined groups: veterans, frontline providers, and managers.

**Figure 2:**
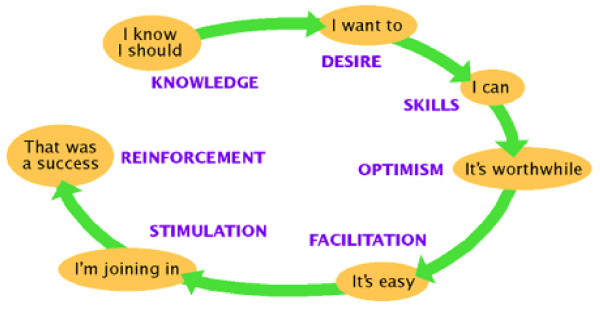
**Sequential model of behavioral and social change**. **Source**: Robinson, L, "The Seven Doors Social Marketing Approach," 1998. https://www.comminit.com/en/node/201090 Accessed 16 September 2009.

Robinson proposes a seven-step sequential process through which social marketing can affect behavior change (see Figure 2) [34]. This process can be illustrated by the example of how social marketing could encourage veterans to obtain treatment for depression. The first step is providing the individuals whose behavior the marketer seeks to change with *knowledge *regarding the beneficial behavior. For example, a depression care program might inform veterans that they should seek treatment if they are depressed because it will improve their overall quality of life. Marketing messages also can help instill the *desire *to adopt the new behavior, for example, by convincing depressed veterans that seeking treatment will enhance the time they spend with their spouses, children, and grandchildren.

The next step is providing *skill sets and resources *that enable the individual to make the behavior change. These might include access to educational materials, antidepressant medications, and depression care managers. The marketer must then demonstrate real or potential outcomes of adopting the new behavior (e.g., feeling down in the dumps less often and enjoying life experiences more), so as to instill a sense of *optimism*.

The next step, *facilitation*, helps convince the audience that it is feasible, even easy, to adopt the new behavior. Social modeling is one mechanism for achieving this goal [35,36]. Social models are individuals similar to the audience who model successful execution of the new behavior. When individuals view those who are similar executing a behavior, it enhances their self-efficacy (i.e., confidence) for performing that behavior themselves. And high self-efficacy increases the likelihood that the target individuals also will execute the behavior [35,36]. Thus, for example, meeting peers who have successfully completed depression treatment can convince veterans that their own depression is treatable as well.

Having demonstrated that the behavior change is feasible, the social marketer must make use of *stimuli *to create action. For example, the target individuals should now take concrete steps to change their behavior (e.g., taking antidepressant medications as prescribed). Finally, during the *reinforcement *step, marketing closes the loop by feeding back positive outcomes that help individuals take further steps to form a new habit. Evidence indicates that a "mastery experience," such as confronting a challenging work or family situation without becoming depressed, enhances the likelihood that a newly adopted behavior will continue [35,36]. The reinforcement step also creates potential "sales representatives" who can be social models for their peers.

Table 3 provides examples of how social marketing messages that support a depression care program could be targeted to the different VHA audience segments of veterans, providers, and managers.

**Table 3:** Social marketing approach to effecting behavior change in Veterans Health Administration market segments.

Stage of behavior change	Social marketing objectives	Sample messages
**Knowledge:** "I know I should"	Provide target audience with knowledge about the desired course of action:	
	• Manager: Support the new depression care program;	• Manager; "I know I should support this program."
	• Provider: Utilize the program; and	• Provider: "I know I should refer my depressed patients to this program."
	• Veteran: Seek treatment for depression.	• Veteran: "I know I should get treated if I'm depressed."

**Desire:** "I want to"	Create desire by presenting image of benefits of new behavior:	
	• Manager: Better patient outcomes and potential for additional funding;	• Manager: "I want to enhance my facility's reputation and obtain additional funding."
	• Provider: Increased patient compliance with depression treatment; and	• Provider: "I want my patients to have improved mood."
	• Veteran: What life could look like after depression is treated.	• Veteran: "I want to enjoy my life more fully."

**Skills and resources:** "I can"	Provide skill set, tools, and resources to enable implementation of desired course of action:	
	• Manager: Resources to implement the depression care program;	• Manager; "I know how to implement this program."
	• Provider: Tools and staff support to utilize the program; and	• Provider: "I know how to refer my patients to this program."
	• Veteran: Facilitated access to the program.	• Veteran: "I know how to get treatment if I'm depressed."

**Optimism:**"It's worthwhile"	Demonstrate real or potential outcomes related to desired course of action:	
	• Manager: Depression care program improves outcomes for a reasonable cost;	• Manager: "The program's quality or financial benefits are worth the cost."
	• Provider: Patients have improved mood and are healthier; and	• Provider: "My depressed patients are getting better."
	• Veteran: Quality of life improves.	• Veteran: "I feel better."

**Facilitation:** "It's easy"	Demonstrate the desired behavior is feasible	
	• All segments: Enhance self-efficacy for implementing, utilizing, or accessing the program (e.g. through social modeling); and testimonials from similar others about their positive experiences.	• All segments: "I am like you, and this depression care program works for me. It can work for you."

**Stimulation:** "I'm joining in"	Use stimuli to create action, i.e., adopt new behavior:	
	• Manager: Primary care clinics are beginning to refer patients to the program;	• Manager: "We're seeing benefits from this program."
	• Provider: Patients begin to comply with depression treatment; and	• Provider: "I'm learning how to utilize the program."
	• Veteran: Small steps toward improved mood.	• Veteran: "I haven't felt really down in a week."

**Reinforcement:**	Show that positive outcome will help individuals form new personal habits	
	• All segments: Personal mastery experiences accumulate; and feedback from managers, providers, and veterans regarding their progress.	• Manager: "We're making the depression care program work." • Provider: "I routinely refer my depressed patients to this program." • • Veteran: "I'm not letting depression control my life."

## A social marketing approach to promote the TIDES collaborative depression care model in VHA

Once the first-generation TIDES sites had developed and demonstrated a safe, acceptable, VHA-adapted collaborative care model with high fidelity to the published evidence, the next challenges MH-QUERI faced were replicating the model effectively at additional sites, and obtaining support and resources from national and regional leadership to disseminate TIDES nationally. By 2004, the TIDES team had used evidence-based quality improvement (EBQI) methods to develop and test, through Plan-Do-Study-Act (PDSA) cycles, a VA-adapted collaborative care model that could be sustained as part of routine care in VA [37]. In 2005, the TIDES team began working with university-based experts in health care management (JL and FH) as part of a VHA Health Services Research and Development (HSR&D) Service implementation research grant to identify approaches to accelerate spread of the model. During this collaboration, it became clear that a social marketing approach could further leverage the expertise of the TIDES team and the knowledge gained at the first-generation sites.

Below we first outline the EBQI method used by the TIDES team, and then describe how the social marketing approach informed TIDES national dissemination efforts. We next outline how social marketing theory and qualitative evaluation findings were used to target marketing messages to different VHA audience segments, and describe the TIDES marketing activities directed at each of those segments. Finally, we briefly discuss evaluation of the marketing efforts as part of the ongoing evaluation of TIDES dissemination.

### Evidence-based quality improvement

TIDES implementation strives to balance two competing objectives: standardizing across VHA sites the crucial evidence-based elements of the collaborative depression care model, while also allowing sites to customize other elements to meet local conditions and obtain buy-in from clinicians and staff. Grol, Wensing, and Eccles characterize these objectives as rational vs. participation approaches to implementation [28]. Fixsen and colleagues emphasize the importance of knowing the essential core components of an intervention, while recognizing that local implementation is always influenced by the organizational structure and culture of each site [38].

TIDES implementation utilizes an evidence-based quality improvement (EBQI) method to strike the balance between standardization and local customization in the context of a research/clinical partnership [37,39,40]. EBQI begins with a structured rating process involving regional administrative, primary care, nursing and mental health leaders [41]. These leaders rate the feasibility and desirability of specific alternative model features (see rating tool - Additional file 1]. For example, VHA leadership endorsed the feature "electronic support for the model" as essential, while the literature shows both electronic and paper versions as achieving effectiveness. MH-QUERI health services researchers provided guidance on lessons learned from the best available evidence. Local QI teams used leadership guidance on key features to design and implement a VHA-adapted collaborative care model at first generation sites, using sequential PDSA cycles.

In 2005, TIDES began a second set of projects focused on spread of collaborative care to additional regions and medical centers. Social marketing theory guided national spread activities, while the research team continued to provide technical expert support to ongoing PDSA cycles in all sites [22]. After a year of implementation and spread to second generation sites (2006), VA's national Primary Care Mental Health Integration Initiative decided to foster national implementation.

At implementing sites, the EBQI partnership fosters two-way conversations between a research team of technical experts and teams of clinical and management leaders. In these interactions, researchers explain the basis for each element of the TIDES model (direct evidence from research, expert recommendations from researchers, or project team members' experience). Managers, clinicians, and staff at an implementing site explain local issues that must be accounted for in implementing TIDES. Consensus can emerge regarding which elements will be customized, and the research team is able to synthesize local experience from multiple sites to strengthen future implementation efforts. Clinical and clinical management leaders, however, make all final decisions on design and implementation [37].

Consistent with social marketing theory, the TIDES team worked with users to develop and test, through multiple PDSA cycles, a series of ready-to-use tools to assist VISNs and facilities with a clear "blueprint" for implementing TIDES. These include: training and certification materials for depression care managers; computerized referral tools integrated into the VHA Computerized Patient Record System (CPRS); educational materials for primary care physicians, including slides and pocket cards; examination room posters; guidelines for supervising psychiatrists; implementation recommendations/sign-offs for facility managers; and job descriptions and functional statements for care managers. Examples of some of these tools are included here [Additional files 2, 3, 4].

### Marketing as a component of TIDES national dissemination

Health services research at the first-generation sites demonstrated that the TIDES model was effective and cost-effective [42,43]. The EBQI method shows subsequent TIDES sites *what *to implement and *how *to implement it. Nevertheless, the success of TIDES dissemination depends on convincing VHA managers and clinicians *why *it is worthwhile for them to support TIDES. Educational seminars and consultation by the university-based management experts highlighted that a social marketing approach could help achieve this needed behavior change. As a result, the MH-QUERI decided to include social marketing as one of the four major aspects of the TIDES national dissemination plan, with specific measurable goals that are outlined in the discussion of evaluation below [22]. The TIDES team also explicitly identified audience segments and developed marketing messages and materials tailored to different segments.

Most marketing materials were based on data from the first-generation sites and the EBQI process. The crucial insight from marketing was that those data had to be translated into information that would promote behavior changes by managers and clinicians. For example, one of the most effective marketing tools was a carefully edited and designed two-page overview of TIDES that succinctly describes what TIDES is, what it accomplishes, how it works, and the evidence supporting it. That overview is shown here [Additional file 5].

Psychology and social marketing research suggest that individuals find "similar others" to be the most convincing sources of information about behavior change [25,35,44]. Therefore, the TIDES team more explicitly recognized staff from first-generation sites as potential marketing representatives for subsequent sites. These included managers from VISNs and facilities that had successfully implemented TIDES, frontline staff who served as clinical champions for it, and depression care managers. TIDES included leaders from first-generation and second-generation sites as "faculty" in the TIDES program, and engaged them in continued training and spread activities.

### Audience segmentation

Audience segmentation is a crucial marketing task that partitions target customers into different groups based on such factors as their needs and expected responsiveness to the marketing message. Targeting to specific audience segments increases the efficiency and effectiveness of marketing campaigns [8,9]. The TIDES marketing effort has targeted several distinct VHA audience segments, including national leadership, regional (VISN) leadership, facility managers, frontline providers (clinicians), and veterans.

Identifying decision processes, relevant information to include in marketing messages, and effective communication methods for each of these diverse segments is challenging. Table 4 illustrates this challenge by providing examples of how marketing messages promoting implementation of collaborative depression care can be tailored to different VHA audience segments.

**Table 4:** Example social marketing messages for Veterans Health Administration audience segments.

Audience segment	Key information for decision or behavior change	Sample message
VACO** Leaders VISN*** Leaders	Evidence regarding cost and quality impact on the veteran population of adopting the new depression care program.	"I want to facilitate the implementation of this new program at all VHA*** facilities."

**Facility Managers** (Director, chief of staff, chief medical officer, service line directors, primary care director)	Benefits and costs of the new depression care program and proven techniques for implementing it.	"I support the new depression care program and know how to encourage providers to utilize it."

**Frontline Providers **(Primary care and specialty physicians, nurses, pharmacists, other health professionals)	Impact of the new depression care program on veterans' health and clinic workload.	"I know I should refer my patients to the new depression care program, and am able to do so."

**Veterans **(i.e., consumers)	Benefits of recognizing depression and seeking treatment for it	"I know depression can be treated, and I know how I can get that treatment."

*VACO: VA Central Office, senior administrative and clinical leadership in Washington, DC

** VISN: Veterans Integrated Service Network, 21 accountable regional networks

***VHA: Veterans Health Administration

The TIDES team utilized several sources of audience research to craft messages for different audience segments. For example, senior members of MH-QUERI drew upon their own experience to hypothesize how best to market TIDES to the VHA national leadership. The TIDES team extracted lessons about how to market to the VISN and facility segments from its experience with the first-generation TIDES sites, as well as from systematic discussions with VISN leaders and facility managers.

An important source of information for the social marketing effort was systematic collection and analysis of qualitative data following initial TIDES development in first-generation sites. The qualitative data team conducted semi-structured interviews and site visits to explore the perspectives of patients, clinicians, managers, and VA leaders. The team also used evaluation findings to produce empirically-based recommendations for marketing messages targeting the manager and clinician segments. For example, VHA managers and frontline providers utilize somewhat different rationales for choosing to adopt new programs. Whereas managers want to know that programs improve the quality of care in a cost-effective manner (i.e., the "business case" for implementation), providers want to know that programs improve quality without increasing their work burdens [45].

### Targeted marketing to audience segments

To promote implementation of depression collaborative care at multiple sites, and to gain support for its national dissemination, the TIDES team has conducted marketing activities specifically tailored for each of several audience segments: VHA national leadership, regional (VISN) leadership, facility managers, frontline providers, and veterans. For each segment, the social marketing approach has helped to identify the desired decision or behavior change, outline that segment's decision criteria, and suggest how to tailor and deliver marketing messages for that segment.

#### VHA national leadership

The VHA Central Office audience comprises several diverse stakeholder groups, such as VHA's National Leadership Board, the Serious Mental Illness committee, and the committee of VISN Chief Medical Officers. The desired decision by this audience was a national endorsement of collaborative care for depression. Framing its decision criteria from a national perspective, this audience is concerned with such issues as: the importance to VHA of the problem a proposed intervention addresses (depression, in the case of TIDES); the intervention's compatibility with VHA's national strategic plan; and its economic implications. Marketing materials include highly distilled information, such as summaries of research evidence and national-level benefit and cost estimates. TIDES and MH-QUERI leaders used informal techniques such as individual and small group meetings; they also participated in a national mental health strategic planning effort in 2004.

#### Regional leadership

The target audience at a VISN includes its Director and other senior clinical and management leaders, who are often organized into an executive leadership team, a clinical practice council, and a quality improvement council. These groups must decide whether to endorse collaborative care, and whether to support it either centrally or by providing resources and implementation support to individual facilities. VISN-level decision criteria include the prevalence and impact of depression in the VISN population, the benefits to be gained from implementing TIDES, the staff and financial resources required to implement it, and incentives from central office including policies, performance measures, or additional resources. The TIDES team must therefore be able to explain the benefits and costs of TIDES--that is, its "business case" [46]--in comparison to competing initiatives and priorities the VISN is striving to address. A comprehensive set of marketing materials has been created for this audience, including polished promotional brochures to provoke initial interest and a TIDES program Intranet website.

Because there are 21 VISNs, techniques for marketing to them are more formal than at the national level. The TIDES team has utilized a variety of techniques to reach VISN leaders, including: formal solicitations followed by meetings or teleconferences, informal contacts such as those that occur at national meetings of VISN Directors and leaders, testimonials from early-adopter VISNs, and making internal consultants from facilities that have successfully implemented TIDES available to help with problem solving during implementation. Anecdotal evidence suggests that this approach has been effective among regional managers, and some of them have become strong proponents of TIDES. Leaders from VISNs not participating in the early phases of TIDES contacted the TIDES team and asked to be considered for subsequent waves of the national implementation. By 2009, all or nearly all VISNs have accessed TIDES materials, and about half of all VISNs have participated in TIDES training to some level.

#### Facility managers

Facilities that implement TIDES can range from large VA Medical Centers (VAMCs) to freestanding Community-Based Outpatient Clinics (CBOCs). At a VAMC, the target audience would include the director; chief of staff; directors of primary care, mental health, and nursing; and clinic managers. The audience at a CBOC would be the lead clinicians and administrators. At any facility, the desired decision is that the TIDES collaborative care model be implemented. Decision criteria vary widely across facilities, but may include the projected quality of care benefits for the facility's population; the feasibility of implementation, given the resources provided and competing initiatives and mandates; and effects on the facility's workflow and clinical processes. Tools that support the TIDES EBQI process, such as step-by-step implementation plans, training materials, and explanations of clinical evidence, can be used as marketing materials at the facility level. Marketing techniques are more interpersonal than at the VISN level, due to variation in local conditions and decision processes. These techniques include small group meetings and invitations to regional or national conferences for clinical leaders and care managers.

Local customization of the TIDES model is systematically addressed at the outset in a meeting of facility, nursing, primary care, and mental health local leaders. These leaders complete a charter, describing who will be responsible for which TIDES functions. Leaders and researcher technical experts then meet for site implementation training, during which TIDES processes are adapted to local needs. These meetings serve as a pre-implementation venue for marketing key aspects of TIDES as well as an opportunity to engage potential clinician opinion leaders.

Implementation support available from the TIDES team also helps to differentiate TIDES from competing priorities for improvement at the facility level. For example, TIDES provides written and computerized tools for facilities to implement, depression care managers may be centralized at the VISN level to minimize the demands on local facility staffing, and peer managers at other facilities are available for consultation about the challenges and benefits of implementing TIDES.

#### Frontline providers

The TIDES collaborative care model can only succeed if frontline providers in the clinics, especially primary care physicians and nurses, are convinced of its value. The desired action on their part is a behavior change, that is, to refer their patients to depression care managers. Evaluation findings from first-generation sites provide several recommendations about marketing to this audience [45]. Busy providers must be persuaded that referrals to the TIDES care manager will help their patients, and not add an undue burden to their workload. The most effective marketing materials for this audience are practice-oriented; for example, concise, clinically relevant summaries of the evidence; vivid case examples and testimonials; clear procedures; and targeted training coincident in time with TIDES startup. Frontline providers strongly prefer verbal, especially face-to-face, presentation of marketing information over written presentation (e.g., brochures or electronic mail). Finally, they are most likely to find marketing messages convincing when fellow providers, including clinicians like themselves or TIDES care managers, deliver the message.

#### Veterans

TIDES and other care management programs can only improve outcomes if patients enter them and participate actively. TIDES has developed a series of patient education and self-management support materials. Because there is substantial evidence that vivid information (e.g., testimonials or case examples) is more convincing than pallid information (e.g., statistics) [47], these materials include information from veterans who have successfully utilized TIDES. Veterans who have benefited from TIDES also can serve as social models by providing written or in-person testimonials to their peers. Finally, because similar others who are of somewhat higher status than the target audience can be particularly compelling information sources, Veterans Service Organization (VSO) representatives may be effective in helping veterans recognize that depression is a treatable condition and that TIDES is an effective way to get such treatment.

### Evaluating TIDES marketing activities

The overall evaluation of the TIDES national dissemination effort includes some evaluation of marketing activities [22]. For example, 7 of the 18 goals of the TIDES national dissemination plan relate to marketing, and progress toward each can be measured:

1. Develop a TIDES marketing plan.

2. Keep key national stakeholders apprised of TIDES expansion progress.

3. Develop the "business case" for collaborative depression care.

4. Disseminate scientific findings related to TIDES implementation and evaluation.

5. Recruit four new VISNs to implement TIDES by 2007, and 15 more VISNs by 2010.

6. Secure funding to support TIDES spread and sustainability.

7. Develop a cadre of TIDES experts to serve as consultants to VISNs.

Marketing-related goals in the ongoing evaluation of TIDES include measuring the number of new implementing sites and the refinement of TIDES tools for implementing sites. These results are being published separately. Nevertheless, it is challenging to independently evaluate the contribution of the TIDES marketing effort to the overall effectiveness of the TIDES national dissemination effort, for which the main outcome measure is the number of VISNs and VHA facilities that adopt the TIDES depression care model.

Focused evaluation of marketing activities will be conducted as resources permit. This evaluation will emphasize process and effectiveness measures, especially for the VISN, facility, and frontline provider audience segments. For example, at the VISN level process measures would include the number of VISNs solicited and the number of follow-up meetings or inquiries. Process measures also could count the number of facilities contacted by the TIDES team, and the number of providers receiving marketing materials. The effectiveness of marketing activities at the VISN level could be assessed by interviewing VISN leaders to obtain their feedback about how the marketing activities informed and influenced their decision-making. At the facility level, similar interviews could be conducted, both at facilities that adopt TIDES, as well as some that do not. At the provider level, a survey could ask clinicians if they remember receiving the TIDES marketing materials, whether they felt the materials were compelling, and whether they are referring patients to TIDES depression care managers.

## Discussion

Collaborative depression care models have proven effective in improving patient outcomes within and outside VHA. MH-QUERI is engaged in an effort to implement this evidence-based model nationwide, which requires management decision-making at multiple levels of the VHA organization and provider behavior change at hundreds of patient care facilities.

A social marketing approach explicitly informed several TIDES national dissemination activities. Social marketing applies marketing techniques to promote positive behavior change. Although primarily used to promote healthy behaviors in the general population, social marketing can be adapted to promote management, clinician, and patient behavior change in a large integrated healthcare system.

The TIDES model had been extensively evaluated at first-generation sites by an experienced health services research team. This team also used an evidence-based quality improvement (EBQI) method to clarify which elements of the TIDES model should remain standardized and which elements VISNs or facilities could customize to fit local conditions.

Nevertheless, a social marketing perspective allowed the TIDES team to further leverage its expertise and evidence in ways it would not otherwise have done. For example, the TIDES team segmented its target audience into several distinct groups, each with a defined behavior change goal: managers who must decide to implement TIDES and allocate the necessary resources to it, clinicians who refer their patients, and veterans who enroll in the program to treat their depression. The team also utilized its members' experience, as well as qualitative evaluation findings from first-generation TIDES implementation to tailor marketing messages to specific audience segments. MH-QUERI explicitly included measurable marketing goals in its TIDES national dissemination plan.

An important lesson from the TIDES experience is that social marketing efforts should be considered as soon as evidence demonstrating the effectiveness of an intervention becomes available. That way, the research team can help define audience segments crucial for broader implementation, and distill their expertise and data from evaluation of the intervention into marketing materials. Managers and clinicians from early sites also can be recruited as marketing representatives to support implementation at subsequent sites.

The social marketing approach appears applicable to almost any evidence-based intervention, but researchers and QI experts can benefit from consultation with experts in marketing as they embark on using social marketing. For example, marketing experts can help researchers learn to translate often-complex evidence into audience-friendly marketing materials.

However, the marketing activities described above concern one program - TIDES, in one integrated healthcare system - the VHA. The plan is based on mature theory, empirical findings, and the experiences of a very skilled multidisciplinary research team, but positive findings from ongoing evaluation activities will be needed to confirm its generalizability.

## Conclusion

Although social marketing has heretofore been used primarily to promote healthy behaviors among consumer populations, it also appears able to amplify the effectiveness of standard evaluation and implementation techniques. We believe this is one of the first formal applications of social marketing to promote the implementation of an evidence-based intervention among managers and providers in the VHA. Further research by other QUERI centers could explore the applicability of the social marketing approach to facilitating implementation of other interventions, as well as the institutional factors at multiple levels of the VHA that enhance or hinder the effectiveness of social marketing. If such research demonstrates that social marketing can be effective even in a large and internally diverse government agency like the VHA, it also may be an effective approach for promoting implementation of evidence-based interventions in other integrated healthcare systems.

## Competing interests

The authors declare that they have no competing interests.

## Authors' contributions

JL served as a VA Academic Expert in marketing and drafted and revised the manuscript. FH also served as a VA Academic Expert and led the development of the social marketing framework. LP co-led (with JK) the TIDES audience segmentation research. EY and JK are senior members of the TIDES implementation and evaluation teams. LR is TIDES Principal Investigator.

## Supplementary Material

Additional file 1 **Intervention Design Preference Questionnaire**. Questionnaire used as part of EBQI method to solicit regional local VHA leaders' preferences regarding the design of the collaborative depression care model in their region or facility. Click here for file

Additional file 2 **TIDES Care Manager Functional Statement**. Summary of responsibilities and competencies for depression care managers. Click here for file

Additional file 3 **Selected Screen Shots from Initial Assessment Dialog**. Computerized tool used by depression care managers for initial assessment of referred veterans. Click here for file

Additional file 4 **Helping VA Primary Care Providers Manage Patients with Depression**. Summary of TIDES collaborative depression care model from perspective of referring primary care clinician. Click here for file

Additional file 5 **Understanding the TIDES Program**. Two-page overview for marketing purposes that succinctly describes what TIDES is, what it accomplishes, how it works, and the evidence supporting it. Click here for file
