# Supplementary material for: A social marketing approach to implementing evidence-based practice in VHA QUERI: the TIDES depression collaborative care model
Source: Implement Sci. 2009 Sep 28;4:64. doi: 10.1186/1748-5908-4-64 (PMC2762953; doi:10.1186/1748-5908-4-64)
Supplement: Additional file 1 — Intervention Design Preference Questionnaire. Questionnaire used as part of EBQI method to solicit regional local VHA leaders' preferences regarding the design of the collaborative depression care model in their region or facility. [file 1748-5908-4-64-S1.PDF]

MENTAL HEALTH QUERI
Quality Enhancement Research Initiative

Translating Initiatives for Depression into Effective Solutions

TIDES

VISN XX

Intervention Design Preference Questionnaire (IDPQ)

Introduction

TIDES aims to help VISNs improve their depression performance and outcomes. This Intervention Design Preference Questionnaire (IDPQ) is designed to obtain your preferences for initial design specifications for the program at (VAMC or clinic). Your ratings of the importance and feasibility of each component and implementation option will serve to focus the discussion during the Depression Intervention Design panel meeting on XXXXX. Aggregated results will be reported at the meeting and will guide the study team in designing an evidence–based quality improvement intervention for depression care.

Please "point and click" your responses and return the survey as an email attachment to: XXXXXXXXX. If you prefer to work from a hard copy, please fax your completed survey to XXXXXXXXXXX. Thank you.

Before you begin, please enter the information requested below:

Name: ____ **Degree:** ______________

VA Title/Position: ____

Medical Center: ____

HSR&D CENTER OF EXCELLENCE FOR THE STUDY OF HEALTHCARE PROVIDER BEHAVIOR (152)
VA GREATER LOS ANGELES HEALTHCARE SYSTEM (GLA)
SEPULVEDA AMBULATORY CARE CENTER & NURSING HOME
16111 PLUMMER STREET
SEPULVEDA, CA 91343
TEL. (818) 895-9449 FAX (818) 895-5838

TABLE OF CONTENTS

I. SENIOR LEADER INVOLVEMENT 1

II. CLINIC PARTICIPATION 3

III. STAFF PARTICIPATION & SUPERVISION 4

IV. SPECIAL/VULNERABLE POPULATION AND CULTURAL ISSUES 6

V. DETECTION OF PATIENTS WITH DEPRESSION 7

VI. CARE MANAGEMENT 8

VII. MENTAL HEALTH/PRIMARY CARE COLLABORATION 12

VIII. CLINICIAN EDUCATION ON DEPRESSION AND THE TIDES PROJECT 14

IX. ACKNOWLEDGING AND VALUING PROJECT PARTICIPATION 15

X. QUALITY IMPROVEMENT 16

XI. DECISION SUPPORT 18

XII. COMMUNITY OUTREACH 19

TRANSLATING INITIATIVES FOR DEPRESSION INTO EFFECTIVE SOLUTIONS

Intervention Design Preference Questionnaire (IDPQ)

◊ For EACH item below, please mark (x) one box (1-5) to indicate how you rate that item's *importance* to improving depression care in your practice setting ***(Column A)***, and the *feasibility* of implementing that item in your practice setting ***(Column B).***

◊ Please answer every item. If you are unable to rate an item because the meaning is unclear or for another reason, you can so indicate by marking the "?" box.

Please Note:

- An item under the heading "*TIDES Care Model Component*" is a key building block of successful depression improvement interventions.
- Items under the heading "*Implementation Option*" represent possible methods for implementing the specified care model component.

I. Senior Leader Involvement	COLUMN A<br>**How *important* is this item to improving depression care in your practice setting?**<br>Importance Rating<br>Low (1) ⇦ ⇨ High (5) OR ?<br>Check one box for each item	COLUMN B<br>**How *feasible* is it to implement this item in your practice setting?**<br>Feasibility Rating<br>Low (1) ⇦ ⇨ High (5) OR ?<br>Check one box for each item
**A. TIDES Care Model Component**<br>Commitment to depression care improvement by senior leadership is essential to program success.	☐ 1 ☐ 2 ☐ 3 ☐ 4 ☐ 5 or ☐ ?	☐ 1 ☐ 2 ☐ 3 ☐ 4 ☐ 5 or ☐ ?
**1. Implementation Option**<br>Ongoing involvement in this project by the following senior leaders**:**		
**a.** VISN Director	☐ 1 ☐ 2 ☐ 3 ☐ 4 ☐ 5 or ☐ ?	☐ 1 ☐ 2 ☐ 3 ☐ 4 ☐ 5 or ☐ ?
**b.** VISN Medical Director	☐ 1 ☐ 2 ☐ 3 ☐ 4 ☐ 5 or ☐ ?	☐ 1 ☐ 2 ☐ 3 ☐ 4 ☐ 5 or ☐ ?
**c.** VISN Mental Health Specialty Leader	☐ 1 ☐ 2 ☐ 3 ☐ 4 ☐ 5 or ☐ ?	☐ 1 ☐ 2 ☐ 3 ☐ 4 ☐ 5 or ☐ ?
**d.** VISN Primary Care Leader	☐ 1 ☐ 2 ☐ 3 ☐ 4 ☐ 5 or ☐ ?	☐ 1 ☐ 2 ☐ 3 ☐ 4 ☐ 5 or ☐ ?
**e.** VAMC Director	☐ 1 ☐ 2 ☐ 3 ☐ 4 ☐ 5 or ☐ ?	☐ 1 ☐ 2 ☐ 3 ☐ 4 ☐ 5 or ☐ ?
**f.** VAMC Chief of Staff	☐ 1 ☐ 2 ☐ 3 ☐ 4 ☐ 5 or ☐ ?	☐ 1 ☐ 2 ☐ 3 ☐ 4 ☐ 5 or ☐ ?
**g.** VAMC Department of Medicine Chief	☐ 1 ☐ 2 ☐ 3 ☐ 4 ☐ 5 or ☐ ?	☐ 1 ☐ 2 ☐ 3 ☐ 4 ☐ 5 or ☐ ?
**h.** VAMC Ambulatory or Primary Care Chief	☐ 1 ☐ 2 ☐ 3 ☐ 4 ☐ 5 or ☐ ?	☐ 1 ☐ 2 ☐ 3 ☐ 4 ☐ 5 or ☐ ?
**i.** VAMC Mental Health Specialty Chief	☐ 1 ☐ 2 ☐ 3 ☐ 4 ☐ 5 or ☐ ?	☐ 1 ☐ 2 ☐ 3 ☐ 4 ☐ 5 or ☐ ?
**j.** VAMC Nursing Chief	☐ 1 ☐ 2 ☐ 3 ☐ 4 ☐ 5 or ☐ ?	☐ 1 ☐ 2 ☐ 3 ☐ 4 ☐ 5 or ☐ ?

1. **Senior Leader Involvement**	**COLUMN A** **How *important* is this item to improving depression care in your practice setting?** Importance Rating: Low (1) ⇦ ⇨ High (5) OR ? — Check one box for each item	**COLUMN B** **How *feasible* is it to implement this item in your practice setting?** Feasibility Rating: Low (1) ⇦ ⇨ High (5) OR ? — Check one box for each item
**k.** Other (Specify):	☐ 1 ☐ 2 ☐ 3 ☐ 4 ☐ 5 or ☐ ?	☐ 1 ☐ 2 ☐ 3 ☐ 4 ☐ 5 or ☐ ?
**2. Implementation Option** Participation by at least one senior leader in the following types of activities:		
**a.** Review intervention plans and ongoing project results	☐ 1 ☐ 2 ☐ 3 ☐ 4 ☐ 5 or ☐ ?	☐ 1 ☐ 2 ☐ 3 ☐ 4 ☐ 5 or ☐ ?
**b.** Participate in the project's list-serve	☐ 1 ☐ 2 ☐ 3 ☐ 4 ☐ 5 or ☐ ?	☐ 1 ☐ 2 ☐ 3 ☐ 4 ☐ 5 or ☐ ?
**c.** Give awards or support letters to successful project participants	☐ 1 ☐ 2 ☐ 3 ☐ 4 ☐ 5 or ☐ ?	☐ 1 ☐ 2 ☐ 3 ☐ 4 ☐ 5 or ☐ ?
**d.** Authorize release time for project participants	☐ 1 ☐ 2 ☐ 3 ☐ 4 ☐ 5 or ☐ ?	☐ 1 ☐ 2 ☐ 3 ☐ 4 ☐ 5 or ☐ ?
**e.** Participate in quarterly senior leader conference calls	☐ 1 ☐ 2 ☐ 3 ☐ 4 ☐ 5 or ☐ ?	☐ 1 ☐ 2 ☐ 3 ☐ 4 ☐ 5 or ☐ ?
**f.** Other (Specify):	☐ 1 ☐ 2 ☐ 3 ☐ 4 ☐ 5 or ☐ ?	☐ 1 ☐ 2 ☐ 3 ☐ 4 ☐ 5 or ☐ ?

Comments related to items in this section:

II. **Clinic Participation**	**COLUMN A** **How *important* is this item to improving depression care in your practice setting?** Importance Rating: Low (1) ⇦ ⇨ High (5) OR ?	**COLUMN B** **How *feasible* is it to implement this item in your practice setting?** Feasibility Rating: Low (1) ⇦ ⇨ High (5) OR ?
**A. TIDES Care Model Component** Existing TIDES clinics are mostly in mid-sized CBOCs, but VISNs are preparing to expand to large medical centers and underserved areas.	☐1 ☐2 ☐3 ☐4 ☐5 or ☐?	☐1 ☐2 ☐3 ☐4 ☐5 or ☐?
**1. Implementation Option** Which clinics should implement collaborative care management for depression?		
**a.** Medical Centers	☐1 ☐2 ☐3 ☐4 ☐5 or ☐?	☐1 ☐2 ☐3 ☐4 ☐5 or ☐?
**b.** CBOCs with mental health	☐1 ☐2 ☐3 ☐4 ☐5 or ☐?	☐1 ☐2 ☐3 ☐4 ☐5 or ☐?
**c.** CBOCs without mental health	☐1 ☐2 ☐3 ☐4 ☐5 or ☐?	☐1 ☐2 ☐3 ☐4 ☐5 or ☐?
**d.** Contract CBOCs (with or without mental health)	☐1 ☐2 ☐3 ☐4 ☐5 or ☐?	☐1 ☐2 ☐3 ☐4 ☐5 or ☐?
**e.** Other (specify):	☐1 ☐2 ☐3 ☐4 ☐5 or ☐?	☐1 ☐2 ☐3 ☐4 ☐5 or ☐?
**2. Implementation Option** Which clinic personnel should participate in national TIDES working groups?		
**a.** Primary Care leaders	☐1 ☐2 ☐3 ☐4 ☐5 or ☐?	☐1 ☐2 ☐3 ☐4 ☐5 or ☐?
**b.** Mental Health leaders	☐1 ☐2 ☐3 ☐4 ☐5 or ☐?	☐1 ☐2 ☐3 ☐4 ☐5 or ☐?
**c.** Depression Care Managers	☐1 ☐2 ☐3 ☐4 ☐5 or ☐?	☐1 ☐2 ☐3 ☐4 ☐5 or ☐?
**d.** Nursing supervisors	☐1 ☐2 ☐3 ☐4 ☐5 or ☐?	☐1 ☐2 ☐3 ☐4 ☐5 or ☐?
**e.** Quality Management leaders	☐1 ☐2 ☐3 ☐4 ☐5 or ☐?	☐1 ☐2 ☐3 ☐4 ☐5 or ☐?
**f.** IRM personnel	☐1 ☐2 ☐3 ☐4 ☐5 or ☐?	☐1 ☐2 ☐3 ☐4 ☐5 or ☐?
**g.** Other (specify):	☐1 ☐2 ☐3 ☐4 ☐5 or ☐?	☐1 ☐2 ☐3 ☐4 ☐5 or ☐?

Comments related to items in this section:

III. Staff Participation & Supervision	COLUMN A<br>**How *important* is this item to improving depression care in your practice setting?**<br>Importance Rating<br>Low (1) ⇦ ⇨ High (5) OR ?	COLUMN B<br>**How *feasible* is it to implement this item in your practice setting?**<br>Feasibility Rating<br>Low (1) ⇦ ⇨ High (5) OR ?
**A. TIDES Care Model Component**<br>Depression Care Management does not involve providing psychotherapy but does require an affinity for dealing with mental health issues and for working collaboratively with primary care and mental health clinicians.	☐ 1 ☐ 2 ☐ 3 ☐ 4 ☐ 5 or ☐ ?	☐ 1 ☐ 2 ☐ 3 ☐ 4 ☐ 5 or ☐ ?
**1. Implementation Option**<br>Who should do care management?		
**a.** LVN or NA	☐ 1 ☐ 2 ☐ 3 ☐ 4 ☐ 5 or ☐ ?	☐ 1 ☐ 2 ☐ 3 ☐ 4 ☐ 5 or ☐ ?
**b.** RN	☐ 1 ☐ 2 ☐ 3 ☐ 4 ☐ 5 or ☐ ?	☐ 1 ☐ 2 ☐ 3 ☐ 4 ☐ 5 or ☐ ?
**c.** NP	☐ 1 ☐ 2 ☐ 3 ☐ 4 ☐ 5 or ☐ ?	☐ 1 ☐ 2 ☐ 3 ☐ 4 ☐ 5 or ☐ ?
**d.** Social Worker	☐ 1 ☐ 2 ☐ 3 ☐ 4 ☐ 5 or ☐ ?	☐ 1 ☐ 2 ☐ 3 ☐ 4 ☐ 5 or ☐ ?
**e.** Other (specify):	☐ 1 ☐ 2 ☐ 3 ☐ 4 ☐ 5 or ☐ ?	☐ 1 ☐ 2 ☐ 3 ☐ 4 ☐ 5 or ☐ ?
**2. Implementation Option**<br>Where will care managers sit organizationally and physically?		
**a.** Primary Care	☐ 1 ☐ 2 ☐ 3 ☐ 4 ☐ 5 or ☐ ?	☐ 1 ☐ 2 ☐ 3 ☐ 4 ☐ 5 or ☐ ?
**b.** Mental Health	☐ 1 ☐ 2 ☐ 3 ☐ 4 ☐ 5 or ☐ ?	☐ 1 ☐ 2 ☐ 3 ☐ 4 ☐ 5 or ☐ ?
**c.** Telephone Triage	☐ 1 ☐ 2 ☐ 3 ☐ 4 ☐ 5 or ☐ ?	☐ 1 ☐ 2 ☐ 3 ☐ 4 ☐ 5 or ☐ ?
**d.** Other (specify):	☐ 1 ☐ 2 ☐ 3 ☐ 4 ☐ 5 or ☐ ?	☐ 1 ☐ 2 ☐ 3 ☐ 4 ☐ 5 or ☐ ?
**3. Implementation Option**<br>How should care managers be administered?		
**a.** Nurse care managers have cross coverage for absences and vacations	☐ 1 ☐ 2 ☐ 3 ☐ 4 ☐ 5 or ☐ ?	☐ 1 ☐ 2 ☐ 3 ☐ 4 ☐ 5 or ☐ ?
**b.** Care managers are viewed as a VISN level resource	☐ 1 ☐ 2 ☐ 3 ☐ 4 ☐ 5 or ☐ ?	☐ 1 ☐ 2 ☐ 3 ☐ 4 ☐ 5 or ☐ ?
**c.** Care managers are viewed as medical center resources	☐ 1 ☐ 2 ☐ 3 ☐ 4 ☐ 5 or ☐ ?	☐ 1 ☐ 2 ☐ 3 ☐ 4 ☐ 5 or ☐ ?
**d.** Other (specify):	☐ 1 ☐ 2 ☐ 3 ☐ 4 ☐ 5 or ☐ ?	☐ 1 ☐ 2 ☐ 3 ☐ 4 ☐ 5 or ☐ ?
**B. TIDES Care Model Component**<br>Nurse Care Managers are trained to carry out assessment, patient self-management support,	☐ 1 ☐ 2 ☐ 3 ☐ 4 ☐ 5 or ☐ ?	☐ 1 ☐ 2 ☐ 3 ☐ 4 ☐ 5 or ☐ ?

III. **Staff Participation & Supervision**	COLUMN A<br>**How *important* is this item to improving depression care in your practice setting?**<br>Importance Rating<br>Low (1) ⇦ ⇨ High (5) OR ?	COLUMN B<br>**How *feasible* is it to implement this item in your practice setting?**<br>Feasibility Rating<br>Low (1) ⇦ ⇨ High (5) OR ?
and care management using project protocols. The EES is developing care manager training material and certification, but there needs to be an ongoing means of monitoring staff training locally.		
**1. Implementation Option**<br>Who will be responsible for orienting, training and retraining care managers?		
**a.** Mental Health	☐ 1 ☐ 2 ☐ 3 ☐ 4 ☐ 5 or ☐ ?	☐ 1 ☐ 2 ☐ 3 ☐ 4 ☐ 5 or ☐ ?
**b.** Primary Care	☐ 1 ☐ 2 ☐ 3 ☐ 4 ☐ 5 or ☐ ?	☐ 1 ☐ 2 ☐ 3 ☐ 4 ☐ 5 or ☐ ?
**c.** Nurse Executive	☐ 1 ☐ 2 ☐ 3 ☐ 4 ☐ 5 or ☐ ?	☐ 1 ☐ 2 ☐ 3 ☐ 4 ☐ 5 or ☐ ?
**d.** Other (specify):	☐ 1 ☐ 2 ☐ 3 ☐ 4 ☐ 5 or ☐ ?	☐ 1 ☐ 2 ☐ 3 ☐ 4 ☐ 5 or ☐ ?

Comments related to items in this section:

	COLUMN A<br>**How *important* is this item to improving depression care in your practice setting?**	COLUMN B<br>**How *feasible* is it to implement this item in your practice setting?**
	Importance Rating	Feasibility Rating
**IV. Special/Vulnerable Population and Cultural Issues**	Low (1) ⇦ ⇨ High (5) OR ?	Low (1) ⇦ ⇨ High (5) OR ?
	Check one box for each item	Check one box for each item

A. TIDES Care Model Component
Outreach/adaptation of intervention materials, procedures, and education to accommodate key special or vulnerable populations.

1. Implementation Option
Importance of outreach/adaptations to accommodate each of the following:

Item	Column A	Column B
**a.** Iraqi Freedom returnees	☐1 ☐2 ☐3 ☐4 ☐5 or ☐?	☐1 ☐2 ☐3 ☐4 ☐5 or ☐?
**b.** Geriatric patients	☐1 ☐2 ☐3 ☐4 ☐5 or ☐?	☐1 ☐2 ☐3 ☐4 ☐5 or ☐?
**c.** African Americans	☐1 ☐2 ☐3 ☐4 ☐5 or ☐?	☐1 ☐2 ☐3 ☐4 ☐5 or ☐?
**d.** Asian Americans	☐1 ☐2 ☐3 ☐4 ☐5 or ☐?	☐1 ☐2 ☐3 ☐4 ☐5 or ☐?
**e.** American Indians	☐1 ☐2 ☐3 ☐4 ☐5 or ☐?	☐1 ☐2 ☐3 ☐4 ☐5 or ☐?
**f.** Hispanics	☐1 ☐2 ☐3 ☐4 ☐5 or ☐?	☐1 ☐2 ☐3 ☐4 ☐5 or ☐?
**g.** Women	☐1 ☐2 ☐3 ☐4 ☐5 or ☐?	☐1 ☐2 ☐3 ☐4 ☐5 or ☐?
**h.** Patients who live far from their source of care	☐1 ☐2 ☐3 ☐4 ☐5 or ☐?	☐1 ☐2 ☐3 ☐4 ☐5 or ☐?
**i.** Other (Specify):	☐1 ☐2 ☐3 ☐4 ☐5 or ☐?	☐1 ☐2 ☐3 ☐4 ☐5 or ☐?
**j.** Other (Specify):	☐1 ☐2 ☐3 ☐4 ☐5 or ☐?	☐1 ☐2 ☐3 ☐4 ☐5 or ☐?

Comments related to items in this section:

	COLUMN A<br>**How *important* is this item to improving depression care in your practice setting?**<br>Importance Rating<br>Low (1) ⇦ ⇨ High (5) OR ?<br>Check one box for each item	COLUMN B<br>**How *feasible* is it to implement this item in your practice setting?**<br>Feasibility Rating<br>Low (1) ⇦ ⇨ High (5) OR ?<br>Check one box for each item
V. **Detection of Patients with Depression,**		
**A. TIDES Care Model Component**<br>An active process for detecting patients with depression is needed.	☐ 1 ☐ 2 ☐ 3 ☐ 4 ☐ 5 or ☐ ?	☐ 1 ☐ 2 ☐ 3 ☐ 4 ☐ 5 or ☐ ?
**1. Implementation Option**<br>Detecting patients with:		
a. Major depression	☐ 1 ☐ 2 ☐ 3 ☐ 4 ☐ 5 or ☐ ?	☐ 1 ☐ 2 ☐ 3 ☐ 4 ☐ 5 or ☐ ?
b. Dysthymia	☐ 1 ☐ 2 ☐ 3 ☐ 4 ☐ 5 or ☐ ?	☐ 1 ☐ 2 ☐ 3 ☐ 4 ☐ 5 or ☐ ?
c. Minor depression with past history	☐ 1 ☐ 2 ☐ 3 ☐ 4 ☐ 5 or ☐ ?	☐ 1 ☐ 2 ☐ 3 ☐ 4 ☐ 5 or ☐ ?
d. Minor depression without past history	☐ 1 ☐ 2 ☐ 3 ☐ 4 ☐ 5 or ☐ ?	☐ 1 ☐ 2 ☐ 3 ☐ 4 ☐ 5 or ☐ ?
e. Other (Specify):	☐ 1 ☐ 2 ☐ 3 ☐ 4 ☐ 5 or ☐ ?	☐ 1 ☐ 2 ☐ 3 ☐ 4 ☐ 5 or ☐ ?
**2. Implementation Option**<br>A screening program for patients visiting general medical clinics that**:**		
a. Is included in the CPRS reminder system	☐ 1 ☐ 2 ☐ 3 ☐ 4 ☐ 5 or ☐ ?	☐ 1 ☐ 2 ☐ 3 ☐ 4 ☐ 5 or ☐ ?
b. Is carried out by primary care staff (e.g.: receptionists, clerks, nurses, or nursing assistants)	☐ 1 ☐ 2 ☐ 3 ☐ 4 ☐ 5 or ☐ ?	☐ 1 ☐ 2 ☐ 3 ☐ 4 ☐ 5 or ☐ ?
c. Is carried out by primary care clinicians (NP's, PA's, or MD's)	☐ 1 ☐ 2 ☐ 3 ☐ 4 ☐ 5 or ☐ ?	☐ 1 ☐ 2 ☐ 3 ☐ 4 ☐ 5 or ☐ ?
d. Can be carried out by mail or telephone	☐ 1 ☐ 2 ☐ 3 ☐ 4 ☐ 5 or ☐ ?	☐ 1 ☐ 2 ☐ 3 ☐ 4 ☐ 5 or ☐ ?
e. Other (Specify):	☐ 1 ☐ 2 ☐ 3 ☐ 4 ☐ 5 or ☐ ?	☐ 1 ☐ 2 ☐ 3 ☐ 4 ☐ 5 or ☐ ?
**3. Implementation Option**<br>Patients screening positive must be assessed further by:		
a. Primary care clinicians	☐ 1 ☐ 2 ☐ 3 ☐ 4 ☐ 5 or ☐ ?	☐ 1 ☐ 2 ☐ 3 ☐ 4 ☐ 5 or ☐ ?
b. Mental health specialists	☐ 1 ☐ 2 ☐ 3 ☐ 4 ☐ 5 or ☐ ?	☐ 1 ☐ 2 ☐ 3 ☐ 4 ☐ 5 or ☐ ?
c. Qualified depression care managers	☐ 1 ☐ 2 ☐ 3 ☐ 4 ☐ 5 or ☐ ?	☐ 1 ☐ 2 ☐ 3 ☐ 4 ☐ 5 or ☐ ?
d. Other (Specify):	☐ 1 ☐ 2 ☐ 3 ☐ 4 ☐ 5 or ☐ ?	☐ 1 ☐ 2 ☐ 3 ☐ 4 ☐ 5 or ☐ ?

Comments related to items in this section:

	COLUMN A<br>How important is this item to improving depression care in your practice setting?	COLUMN B<br>How feasible is it to implement this item in your practice setting?
	**Importance Rating**	**Feasibility Rating**
	Low (1) ⇦ ⇨ High (5) OR ?	Low (1) ⇦ ⇨ High (5) OR ?
**VI. Care Management,**	Check one box for each item	Check one box for each item
**A. TIDES Care Model Component**<br>Screening. All patients screening positive for depression and referred to the depression care manager are assessed for diagnosis and co-morbidity (20 min.) and given patient education and activation to promote self-management (20 min.).	☐1 ☐2 ☐3 ☐4 ☐5 or ☐?	☐1 ☐2 ☐3 ☐4 ☐5 or ☐?
**1. Implementation Option**<br>Results of initial clinical assessment are provided to:		
a. Primary care clinician	☐1 ☐2 ☐3 ☐4 ☐5 or ☐?	☐1 ☐2 ☐3 ☐4 ☐5 or ☐?
b. Mental health specialist	☐1 ☐2 ☐3 ☐4 ☐5 or ☐?	☐1 ☐2 ☐3 ☐4 ☐5 or ☐?
c. Other (Specify):	☐1 ☐2 ☐3 ☐4 ☐5 or ☐?	☐1 ☐2 ☐3 ☐4 ☐5 or ☐?
**2. Implementation Option**<br>Availability of nurse care manager assessment:		
a. On-site patient assessment and self-management support	☐1 ☐2 ☐3 ☐4 ☐5 or ☐?	☐1 ☐2 ☐3 ☐4 ☐5 or ☐?
b. Off-site patient assessment and self-management support by Videoconference	☐1 ☐2 ☐3 ☐4 ☐5 or ☐?	☐1 ☐2 ☐3 ☐4 ☐5 or ☐?
c. Off-site patient assessment and self-management support by telephone/mail	☐1 ☐2 ☐3 ☐4 ☐5 or ☐?	☐1 ☐2 ☐3 ☐4 ☐5 or ☐?
d. Other (Specify):	☐1 ☐2 ☐3 ☐4 ☐5 or ☐?	☐1 ☐2 ☐3 ☐4 ☐5 or ☐?
**B. TIDES Care Model Component**<br>Generating a Care Plan. The depression care manager gives the primary care clinician the assessment results, preferably the same day, along with a care plan form. The primary care clinician and patient generate a care plan that is signed off by the patient, the primary care clinician, and the depression care manager.	☐1 ☐2 ☐3 ☐4 ☐5 or ☐?	☐1 ☐2 ☐3 ☐4 ☐5 or ☐?
**1. Implementation Option**<br>Review of the care plan by mental health specialists for patients with:		
a. Depression only	☐1 ☐2 ☐3 ☐4 ☐5 or ☐?	☐1 ☐2 ☐3 ☐4 ☐5 or ☐?
b. Substance abuse	☐1 ☐2 ☐3 ☐4 ☐5 or ☐?	☐1 ☐2 ☐3 ☐4 ☐5 or ☐?
c. Grief reactions	☐1 ☐2 ☐3 ☐4 ☐5 or ☐?	☐1 ☐2 ☐3 ☐4 ☐5 or ☐?

	COLUMN A<br>How important is this item to improving depression care in your practice setting?	COLUMN B<br>How feasible is it to implement this item in your practice setting?
	Importance Rating	Feasibility Rating
	Low (1) ⇦ ⇨ High (5) OR ?	Low (1) ⇦ ⇨ High (5) OR ?
VI. **Care Management,**	Check one box for each item	Check one box for each item
**d.** PTSD	☐1 ☐2 ☐3 ☐4 ☐5 or ☐?	☐1 ☐2 ☐3 ☐4 ☐5 or ☐?
**e.** Serious psychiatric symptoms (e.g., mania, psychosis)	☐1 ☐2 ☐3 ☐4 ☐5 or ☐?	☐1 ☐2 ☐3 ☐4 ☐5 or ☐?
**f.** Past history or current sx of schizophrenia	☐1 ☐2 ☐3 ☐4 ☐5 or ☐?	☐1 ☐2 ☐3 ☐4 ☐5 or ☐?
**g.** Elderly	☐1 ☐2 ☐3 ☐4 ☐5 or ☐?	☐1 ☐2 ☐3 ☐4 ☐5 or ☐?
**h.** History of domestic or other violence or criminal behavior as a perpetrator	☐1 ☐2 ☐3 ☐4 ☐5 or ☐?	☐1 ☐2 ☐3 ☐4 ☐5 or ☐?
**i.** History of physical or sexual assault as a victim	☐1 ☐2 ☐3 ☐4 ☐5 or ☐?	☐1 ☐2 ☐3 ☐4 ☐5 or ☐?
**j.** Other (Specify):	☐1 ☐2 ☐3 ☐4 ☐5 or ☐?	☐1 ☐2 ☐3 ☐4 ☐5 or ☐?
**k.** Other (Specify):	☐1 ☐2 ☐3 ☐4 ☐5 or ☐?	☐1 ☐2 ☐3 ☐4 ☐5 or ☐?
**2. Implementation Option**<br>Should the care manager make suggestions for initial antidepressant treatment choice based on an algorithm?		
**a.** Care manager will use the existing Long Beach or GLA algorithm for initial choice of antidepressants	☐1 ☐2 ☐3 ☐4 ☐5 or ☐?	☐1 ☐2 ☐3 ☐4 ☐5 or ☐?
**b.** A more complex algorithm (TMAP, for instance) is used to make adjustments if initial treatment doesn't work.	☐1 ☐2 ☐3 ☐4 ☐5 or ☐?	☐1 ☐2 ☐3 ☐4 ☐5 or ☐?
**c.** Other (Specify):	☐1 ☐2 ☐3 ☐4 ☐5 or ☐?	☐1 ☐2 ☐3 ☐4 ☐5 or ☐?
**C. TIDES Care Model Component**<br><u>Management</u>. Depressed patients whose care plan indicates antidepressant medication are followed by a nurse care manager for six months, with calls or visits at 1 week, 2 weeks, 4 to 6 weeks, 10 – 12 weeks, 16 weeks, 3 months, and 6 months. Self-management support is integrated into all patient contacts.	☐1 ☐2 ☐3 ☐4 ☐5 or ☐?	☐1 ☐2 ☐3 ☐4 ☐5 or ☐?
**1. Implementation Option**<br>Availability of care manager follow-up contacts:		
**a.** On-site care management	☐1 ☐2 ☐3 ☐4 ☐5 or ☐?	☐1 ☐2 ☐3 ☐4 ☐5 or ☐?
**b.** Off-site care management by telephone/mail	☐1 ☐2 ☐3 ☐4 ☐5 or ☐?	☐1 ☐2 ☐3 ☐4 ☐5 or ☐?
**c.** Off-site care management by videoconference	☐1 ☐2 ☐3 ☐4 ☐5 or ☐?	☐1 ☐2 ☐3 ☐4 ☐5 or ☐?

VI. Care Management,	COLUMN A<br>How important is this item to improving depression care in your practice setting?<br>Importance Rating<br>Low (1) ⇦ ⇨ High (5) OR ?<br>Check one box for each item	COLUMN B<br>How feasible is it to implement this item in your practice setting?<br>Feasibility Rating<br>Low (1) ⇦ ⇨ High (5) OR ?<br>Check one box for each item
**d.** Other (Specify):	☐1 ☐2 ☐3 ☐4 ☐5 or ☐?	☐1 ☐2 ☐3 ☐4 ☐5 or ☐?
**2. Implementation Option**<br>Cases followed by the nurse care manager are reviewed at weekly or bi-weekly intervals by:		
**a.** Mental health specialist Depression Expert Leader	☐1 ☐2 ☐3 ☐4 ☐5 or ☐?	☐1 ☐2 ☐3 ☐4 ☐5 or ☐?
**b.** Primary care Depression Expert Leader	☐1 ☐2 ☐3 ☐4 ☐5 or ☐?	☐1 ☐2 ☐3 ☐4 ☐5 or ☐?
**c.** Quality and Case Management Depression Expert Leader	☐1 ☐2 ☐3 ☐4 ☐5 or ☐?	☐1 ☐2 ☐3 ☐4 ☐5 or ☐?
**d.** Local medical center mental health specialist	☐1 ☐2 ☐3 ☐4 ☐5 or ☐?	☐1 ☐2 ☐3 ☐4 ☐5 or ☐?
**e.** Local medical center primary care clinician	☐1 ☐2 ☐3 ☐4 ☐5 or ☐?	☐1 ☐2 ☐3 ☐4 ☐5 or ☐?
**f.** Psychologically or behaviorally trained nurse supervisor	☐1 ☐2 ☐3 ☐4 ☐5 or ☐?	☐1 ☐2 ☐3 ☐4 ☐5 or ☐?
**g.** Other (Specify):	☐1 ☐2 ☐3 ☐4 ☐5 or ☐?	☐1 ☐2 ☐3 ☐4 ☐5 or ☐?
**3. Implementation Option**<br>Nurse care management availability to patients on antidepressants with comorbid:		
**a.** Substance abuse	☐1 ☐2 ☐3 ☐4 ☐5 or ☐?	☐1 ☐2 ☐3 ☐4 ☐5 or ☐?
**b.** PTSD	☐1 ☐2 ☐3 ☐4 ☐5 or ☐?	☐1 ☐2 ☐3 ☐4 ☐5 or ☐?
**c.** Other (Specify):	☐1 ☐2 ☐3 ☐4 ☐5 or ☐?	☐1 ☐2 ☐3 ☐4 ☐5 or ☐?
**4. Implementation Option**<br>For patients treated with watchful waiting, the nurse case manager calls the patient at one, three, and six months to assess preferences and depression status	☐1 ☐2 ☐3 ☐4 ☐5 or ☐?	☐1 ☐2 ☐3 ☐4 ☐5 or ☐?
**5. Implementation Option**<br>Patient self-management support strategies will include:		
**a.** Websites (TIDES_WAVES, MyHeath*E*Vet)	☐1 ☐2 ☐3 ☐4 ☐5 or ☐?	☐1 ☐2 ☐3 ☐4 ☐5 or ☐?
**b.** Depression Care Manager Support	☐1 ☐2 ☐3 ☐4 ☐5 or ☐?	☐1 ☐2 ☐3 ☐4 ☐5 or ☐?
**c.** Coordinated use of current VAMC resources (groups, peer support)	☐1 ☐2 ☐3 ☐4 ☐5 or ☐?	☐1 ☐2 ☐3 ☐4 ☐5 or ☐?
**d.** Office of Care Coordination technology assisted home-based support	☐1 ☐2 ☐3 ☐4 ☐5 or ☐?	☐1 ☐2 ☐3 ☐4 ☐5 or ☐?

VI. Care Management,

	COLUMN A<br>How important is this item to improving depression care in your practice setting?<br>Importance Rating<br>Low (1) ⇦ ⇨ High (5) OR ?<br>Check one box for each item	COLUMN B<br>How feasible is it to implement this item in your practice setting?<br>Feasibility Rating<br>Low (1) ⇦ ⇨ High (5) OR ?<br>Check one box for each item
**e.** Other (Specify):	☐ 1 ☐ 2 ☐ 3 ☐ 4 ☐ 5 or ☐ ?	☐ 1 ☐ 2 ☐ 3 ☐ 4 ☐ 5 or ☐ ?
**D. TIDES Care Model Component**<br>Liaison with Mental Health. For patients referred to mental health specialty care, nurse care managers ensure that the patient has an appointment and keeps it. The patient is then case-managed by the mental health specialist (i.e., called when they do not show up, monitored at intervals with a symptom index, etc.). The mental health specialist sends a Notification of Therapy Care Plan form back to the nurse and primary care clinician. If/when the patient leaves mental health specialist follow-up, the specialist notifies the nurse and primary care clinician via an exit form.	☐ 1 ☐ 2 ☐ 3 ☐ 4 ☐ 5 or ☐ ?	☐ 1 ☐ 2 ☐ 3 ☐ 4 ☐ 5 or ☐ ?
**1. Implementation Option**<br>Availability of ongoing nurse care management tracking of patients currently being followed by mental health specialists.	☐ 1 ☐ 2 ☐ 3 ☐ 4 ☐ 5 or ☐ ?	☐ 1 ☐ 2 ☐ 3 ☐ 4 ☐ 5 or ☐ ?

Comments related to items in this section:

	COLUMN A<br>How *important* is this item to improving depression care in your practice setting?	COLUMN B<br>How *feasible* is it to implement this item in your practice setting?
	Importance Rating	Feasibility Rating
**VII. Mental Health/Primary Care Collaboration**	Low (1) ⇦ ⇨ High (5) OR ?	Low (1) ⇦ ⇨ High (5) OR ?
	Check one box for each item	Check one box for each item
**A. TIDES Care Model Component**<br>Mental health specialists collaborate with primary care clinicians to ensure high quality depression care.	☐ 1 ☐ 2 ☐ 3 ☐ 4 ☐ 5 or ☐ ?	☐ 1 ☐ 2 ☐ 3 ☐ 4 ☐ 5 or ☐ ?
**1. Implementation Option**<br>Prompt mental health specialist availability for requests from primary care clinicians for:		
**a.** "Curbside" consultation (e.g., doctor-to-doctor in person or by telephone) for non-emergency patient management questions	☐ 1 ☐ 2 ☐ 3 ☐ 4 ☐ 5 or ☐ ?	☐ 1 ☐ 2 ☐ 3 ☐ 4 ☐ 5 or ☐ ?
**b.** Follow-up of "routine" depressed patients on antidepressants	☐ 1 ☐ 2 ☐ 3 ☐ 4 ☐ 5 or ☐ ?	☐ 1 ☐ 2 ☐ 3 ☐ 4 ☐ 5 or ☐ ?
**c.** Follow-up of complex patients or those who have failed initial treatment	☐ 1 ☐ 2 ☐ 3 ☐ 4 ☐ 5 or ☐ ?	☐ 1 ☐ 2 ☐ 3 ☐ 4 ☐ 5 or ☐ ?
**d.** Other (Specify):	☐ 1 ☐ 2 ☐ 3 ☐ 4 ☐ 5 or ☐ ?	☐ 1 ☐ 2 ☐ 3 ☐ 4 ☐ 5 or ☐ ?
**2. Implementation Option:**<br>Assuring prompt access to the following special mental health resources:		
**a.** Geriatrics/geropsychiatry for frail elderly	☐ 1 ☐ 2 ☐ 3 ☐ 4 ☐ 5 or ☐ ?	☐ 1 ☐ 2 ☐ 3 ☐ 4 ☐ 5 or ☐ ?
**b.** Substance abuse program follow-up care for severe substance abuse (incl. dual-disorders)	☐ 1 ☐ 2 ☐ 3 ☐ 4 ☐ 5 or ☐ ?	☐ 1 ☐ 2 ☐ 3 ☐ 4 ☐ 5 or ☐ ?
**c.** Substance abuse program follow-up care for all cases in which ANY comorbid substance abuse is detected	☐ 1 ☐ 2 ☐ 3 ☐ 4 ☐ 5 or ☐ ?	☐ 1 ☐ 2 ☐ 3 ☐ 4 ☐ 5 or ☐ ?
**d.** Grief counseling for recently bereaved patients	☐ 1 ☐ 2 ☐ 3 ☐ 4 ☐ 5 or ☐ ?	☐ 1 ☐ 2 ☐ 3 ☐ 4 ☐ 5 or ☐ ?
**e.** PTSD specialist consultation for patients with symptoms of PTSD	☐ 1 ☐ 2 ☐ 3 ☐ 4 ☐ 5 or ☐ ?	☐ 1 ☐ 2 ☐ 3 ☐ 4 ☐ 5 or ☐ ?
**f.** PTSD program follow-up for patients with severe PTSD	☐ 1 ☐ 2 ☐ 3 ☐ 4 ☐ 5 or ☐ ?	☐ 1 ☐ 2 ☐ 3 ☐ 4 ☐ 5 or ☐ ?
**g.** Other (Specify):	☐ 1 ☐ 2 ☐ 3 ☐ 4 ☐ 5 or ☐ ?	☐ 1 ☐ 2 ☐ 3 ☐ 4 ☐ 5 or ☐ ?
**3. Implementation Option:**<br>What mechanisms are in place to handle emergencies?		
**a.** In-person patient assessment	☐ 1 ☐ 2 ☐ 3 ☐ 4 ☐ 5 or ☐ ?	☐ 1 ☐ 2 ☐ 3 ☐ 4 ☐ 5 or ☐ ?
**b.** Threatened suicide protocol	☐ 1 ☐ 2 ☐ 3 ☐ 4 ☐ 5 or ☐ ?	☐ 1 ☐ 2 ☐ 3 ☐ 4 ☐ 5 or ☐ ?
**c.** Other (Specify):	☐ 1 ☐ 2 ☐ 3 ☐ 4 ☐ 5 or ☐ ?	☐ 1 ☐ 2 ☐ 3 ☐ 4 ☐ 5 or ☐ ?

VII. **Mental Health/Primary Care Collaboration**	**COLUMN A** **How *important* is this item to improving depression care in your practice setting?** Importance Rating: Low (1) ⇦ ⇨ High (5) OR ? — Check one box for each item	**COLUMN B** **How *feasible* is it to implement this item in your practice setting?** Feasibility Rating: Low (1) ⇦ ⇨ High (5) OR ? — Check one box for each item
**B. TIDES Care Model Component** Availability of short-term psychotherapy for patients who prefer psychotherapy, have severe psychosocial stress, or have contraindications to antidepressants.	☐ 1 ☐ 2 ☐ 3 ☐ 4 ☐ 5 or ☐ ?	☐ 1 ☐ 2 ☐ 3 ☐ 4 ☐ 5 or ☐ ?
**1. Implementation Option** Importance of the availability of:		
**a.** Cognitive Behavioral Therapy (CBT) or Interpersonal Therapy (IPT) groups	☐ 1 ☐ 2 ☐ 3 ☐ 4 ☐ 5 or ☐ ?	☐ 1 ☐ 2 ☐ 3 ☐ 4 ☐ 5 or ☐ ?
**b.** CBT or IPT individual sessions	☐ 1 ☐ 2 ☐ 3 ☐ 4 ☐ 5 or ☐ ?	☐ 1 ☐ 2 ☐ 3 ☐ 4 ☐ 5 or ☐ ?
**c.** Vet Center support groups	☐ 1 ☐ 2 ☐ 3 ☐ 4 ☐ 5 or ☐ ?	☐ 1 ☐ 2 ☐ 3 ☐ 4 ☐ 5 or ☐ ?
**d.** Outreach clinics or contracted fee-for-service mental health treatment for veterans in remote areas	☐ 1 ☐ 2 ☐ 3 ☐ 4 ☐ 5 or ☐ ?	☐ 1 ☐ 2 ☐ 3 ☐ 4 ☐ 5 or ☐ ?
**e.** Telemedicine counseling for veterans in remote areas	☐ 1 ☐ 2 ☐ 3 ☐ 4 ☐ 5 or ☐ ?	☐ 1 ☐ 2 ☐ 3 ☐ 4 ☐ 5 or ☐ ?
**f.** Other (Specify):	☐ 1 ☐ 2 ☐ 3 ☐ 4 ☐ 5 or ☐ ?	☐ 1 ☐ 2 ☐ 3 ☐ 4 ☐ 5 or ☐ ?

Comments related to items in this section:

VIII. **Clinician Education on Depression and the TIDES project**	**COLUMN A**<br>**How *important* is this item to improving depression care in your practice setting?**<br>Importance Rating<br>Low (1) ⇦ ⇨ High (5) OR ?<br>Check one box for each item	**COLUMN B**<br>**How *feasible* is it to implement this item in your practice setting?**<br>Feasibility Rating<br>Low (1) ⇦ ⇨ High (5) OR ?<br>Check one box for each item
**A. TIDES Care Model Component**<br>Depression expert leaders are trained and provided with materials (quick reference guides, slides, clinician manuals), and in turn provide seminars, academic detailing, and case review to all primary care providers.	☐1 ☐2 ☐3 ☐4 ☐5 or ☐?	☐1 ☐2 ☐3 ☐4 ☐5 or ☐?
**1. Implementation Option**<br>Education by Depression Expert Leaders for:		
a. Primary care nurses	☐1 ☐2 ☐3 ☐4 ☐5 or ☐?	☐1 ☐2 ☐3 ☐4 ☐5 or ☐?
b. Primary care clinicians	☐1 ☐2 ☐3 ☐4 ☐5 or ☐?	☐1 ☐2 ☐3 ☐4 ☐5 or ☐?
c. Mental health specialists	☐1 ☐2 ☐3 ☐4 ☐5 or ☐?	☐1 ☐2 ☐3 ☐4 ☐5 or ☐?
d. Other (Specify):	☐1 ☐2 ☐3 ☐4 ☐5 or ☐?	☐1 ☐2 ☐3 ☐4 ☐5 or ☐?
**2. Implementation Option**<br>Using the following methods for education:		
a. Grand rounds	☐1 ☐2 ☐3 ☐4 ☐5 or ☐?	☐1 ☐2 ☐3 ☐4 ☐5 or ☐?
b. Seminars/noon conferences	☐1 ☐2 ☐3 ☐4 ☐5 or ☐?	☐1 ☐2 ☐3 ☐4 ☐5 or ☐?
c. Case vignettes	☐1 ☐2 ☐3 ☐4 ☐5 or ☐?	☐1 ☐2 ☐3 ☐4 ☐5 or ☐?
d. Web-based training	☐1 ☐2 ☐3 ☐4 ☐5 or ☐?	☐1 ☐2 ☐3 ☐4 ☐5 or ☐?
e. Academic one-on-one detailing	☐1 ☐2 ☐3 ☐4 ☐5 or ☐?	☐1 ☐2 ☐3 ☐4 ☐5 or ☐?
f. Other (Specify):	☐1 ☐2 ☐3 ☐4 ☐5 or ☐?	☐1 ☐2 ☐3 ☐4 ☐5 or ☐?

Comments related to items in this section:

	COLUMN A<br>**How *important* is this item to improving depression care in your practice setting?**	COLUMN B<br>**How *feasible* is it to implement this item in your practice setting?**
	Importance Rating	Feasibility Rating
	Low (1) ⇦ ⇨ High (5) OR ?	Low (1) ⇦ ⇨ High (5) OR ?
**IX. Acknowledging and Valuing Project Participation**	Check one box for each item	Check one box for each item
**A. TIDES Care Model Component**<br>Authorization of release time and recognition for participants in the depression quality improvement project.	☐ 1 ☐ 2 ☐ 3 ☐ 4 ☐ 5 or ☐ ?	☐ 1 ☐ 2 ☐ 3 ☐ 4 ☐ 5 or ☐ ?
**1. Implementation Option**<br>Prior sign-off on time for project participation by:		
**a.** Medical center leadership	☐ 1 ☐ 2 ☐ 3 ☐ 4 ☐ 5 or ☐ ?	☐ 1 ☐ 2 ☐ 3 ☐ 4 ☐ 5 or ☐ ?
**b.** VISN leadership	☐ 1 ☐ 2 ☐ 3 ☐ 4 ☐ 5 or ☐ ?	☐ 1 ☐ 2 ☐ 3 ☐ 4 ☐ 5 or ☐ ?
**c.** Division or department chief	☐ 1 ☐ 2 ☐ 3 ☐ 4 ☐ 5 or ☐ ?	☐ 1 ☐ 2 ☐ 3 ☐ 4 ☐ 5 or ☐ ?
**d.** Care line/product line chief	☐ 1 ☐ 2 ☐ 3 ☐ 4 ☐ 5 or ☐ ?	☐ 1 ☐ 2 ☐ 3 ☐ 4 ☐ 5 or ☐ ?
**e.** Other (Specify):	☐ 1 ☐ 2 ☐ 3 ☐ 4 ☐ 5 or ☐ ?	☐ 1 ☐ 2 ☐ 3 ☐ 4 ☐ 5 or ☐ ?
**2. Implementation Option**<br>Provision of workload credit for collaborative care activities other than intervention design and implementation (e.g., for curbside consultation, case review) for:		
**a.** Mental health specialists	☐ 1 ☐ 2 ☐ 3 ☐ 4 ☐ 5 or ☐ ?	☐ 1 ☐ 2 ☐ 3 ☐ 4 ☐ 5 or ☐ ?
**b.** Primary care clinicians	☐ 1 ☐ 2 ☐ 3 ☐ 4 ☐ 5 or ☐ ?	☐ 1 ☐ 2 ☐ 3 ☐ 4 ☐ 5 or ☐ ?
**c.** Nurses	☐ 1 ☐ 2 ☐ 3 ☐ 4 ☐ 5 or ☐ ?	☐ 1 ☐ 2 ☐ 3 ☐ 4 ☐ 5 or ☐ ?
**d.** Other (Specify):	☐ 1 ☐ 2 ☐ 3 ☐ 4 ☐ 5 or ☐ ?	☐ 1 ☐ 2 ☐ 3 ☐ 4 ☐ 5 or ☐ ?
**3. Implementation Option**<br>Incorporation of feedback from primary care clinicians into performance evaluations or awards for:		
**a.** Mental health specialists	☐ 1 ☐ 2 ☐ 3 ☐ 4 ☐ 5 or ☐ ?	☐ 1 ☐ 2 ☐ 3 ☐ 4 ☐ 5 or ☐ ?
**b.** Primary care clinicians	☐ 1 ☐ 2 ☐ 3 ☐ 4 ☐ 5 or ☐ ?	☐ 1 ☐ 2 ☐ 3 ☐ 4 ☐ 5 or ☐ ?
**c.** Nurses	☐ 1 ☐ 2 ☐ 3 ☐ 4 ☐ 5 or ☐ ?	☐ 1 ☐ 2 ☐ 3 ☐ 4 ☐ 5 or ☐ ?
**d.** Other (Specify):	☐ 1 ☐ 2 ☐ 3 ☐ 4 ☐ 5 or ☐ ?	☐ 1 ☐ 2 ☐ 3 ☐ 4 ☐ 5 or ☐ ?

Comments related to items in this section:

X. Quality Improvement

	COLUMN A<br>**How *important* is this item to improving depression care in your practice setting?**<br>Importance Rating<br>Low (1) ⇦ ⇨ High (5) OR ?<br>Check one box for each item	COLUMN B<br>**How *feasible* is it to implement this item in your practice setting?**<br>Feasibility Rating<br>Low (1) ⇦ ⇨ High (5) OR ?<br>Check one box for each item
**A. TIDES Care Model Component**<br>The depression improvement project is sponsored and endorsed by VA Office of Quality and Performance.	☐1 ☐2 ☐3 ☐4 ☐5 or ☐?	☐1 ☐2 ☐3 ☐4 ☐5 or ☐?
**1. Intervention Option**<br>Involvement of quality management and IRM personnel to capture workload and performance data:		
a. At the VISN level	☐1 ☐2 ☐3 ☐4 ☐5 or ☐?	☐1 ☐2 ☐3 ☐4 ☐5 or ☐?
b. At the medical center level	☐1 ☐2 ☐3 ☐4 ☐5 or ☐?	☐1 ☐2 ☐3 ☐4 ☐5 or ☐?
c. For documentation of quality improvement for Joint Commission	☐1 ☐2 ☐3 ☐4 ☐5 or ☐?	☐1 ☐2 ☐3 ☐4 ☐5 or ☐?
d. For documentation of improved performance on EPRP review	☐1 ☐2 ☐3 ☐4 ☐5 or ☐?	☐1 ☐2 ☐3 ☐4 ☐5 or ☐?
e. Other (Specify):	☐1 ☐2 ☐3 ☐4 ☐5 or ☐?	☐1 ☐2 ☐3 ☐4 ☐5 or ☐?
**B. TIDES Care Model Component**<br>Nurse care manager assessments of patient symptom severity, adherence to follow-up visits, and adherence to appropriate medication or psychotherapy treatment are captured in the data warehouse and translated into graphs reflecting depression project outcomes (e.g., 90% of patients screening positive for depression will be assessed).	☐1 ☐2 ☐3 ☐4 ☐5 or ☐?	☐1 ☐2 ☐3 ☐4 ☐5 or ☐?
**1. Intervention Option**<br>Regular feedback on program outcomes is provided to:		
a. Network leadership	☐1 ☐2 ☐3 ☐4 ☐5 or ☐?	☐1 ☐2 ☐3 ☐4 ☐5 or ☐?
b. Medical Center leadership	☐1 ☐2 ☐3 ☐4 ☐5 or ☐?	☐1 ☐2 ☐3 ☐4 ☐5 or ☐?
c. Primary Care and Mental Health Leaders	☐1 ☐2 ☐3 ☐4 ☐5 or ☐?	☐1 ☐2 ☐3 ☐4 ☐5 or ☐?
d. Referring clinicians	☐1 ☐2 ☐3 ☐4 ☐5 or ☐?	☐1 ☐2 ☐3 ☐4 ☐5 or ☐?
e. Mental health consultants	☐1 ☐2 ☐3 ☐4 ☐5 or ☐?	☐1 ☐2 ☐3 ☐4 ☐5 or ☐?
f. All clinicians	☐1 ☐2 ☐3 ☐4 ☐5 or ☐?	☐1 ☐2 ☐3 ☐4 ☐5 or ☐?
g. All primary care team members	☐1 ☐2 ☐3 ☐4 ☐5 or ☐?	☐1 ☐2 ☐3 ☐4 ☐5 or ☐?

X. **Quality Improvement**	COLUMN A<br>**How *important* is this item to improving depression care in your practice setting?**<br>Importance Rating<br>Low (1) ⇦ ⇨ High (5) OR ?<br>Check one box for each item	COLUMN B<br>**How *feasible* is it to implement this item in your practice setting?**<br>Feasibility Rating<br>Low (1) ⇦ ⇨ High (5) OR ?<br>Check one box for each item
**h.** Other (Specify):	☐1 ☐2 ☐3 ☐4 ☐5 or ☐?	☐1 ☐2 ☐3 ☐4 ☐5 or ☐?
**2. Intervention Option**<br>**Outcomes will be disseminated**:		
**a.** Via Network newsletter	☐1 ☐2 ☐3 ☐4 ☐5 or ☐?	☐1 ☐2 ☐3 ☐4 ☐5 or ☐?
**b.** By site leaders at medical center educational meetings	☐1 ☐2 ☐3 ☐4 ☐5 or ☐?	☐1 ☐2 ☐3 ☐4 ☐5 or ☐?
**c.** In VA and non-VA publications	☐1 ☐2 ☐3 ☐4 ☐5 or ☐?	☐1 ☐2 ☐3 ☐4 ☐5 or ☐?

Comments related to items in this section:

XI. **Decision Support**	**COLUMN A** **How *important* is this item to improving depression care in your practice setting?** Importance Rating: Low (1) ⇦ ⇨ High (5) OR ? — Check one box for each item	**COLUMN B** **How *feasible* is it to implement this item in your practice setting?** Feasibility Rating: Low (1) ⇦ ⇨ High (5) OR ? — Check one box for each item
**A. TIDES Care Model Component** Nurse assessment, education and case management is supported by VistA/CPRS-imbedded baseline assessment and follow-up protocols.	☐ 1 ☐ 2 ☐ 3 ☐ 4 ☐ 5 or ☐ ?	☐ 1 ☐ 2 ☐ 3 ☐ 4 ☐ 5 or ☐ ?
**1. Implementation Option** Importance/feasibility of:		
**a.** Active involvement of IRM and CAC personnel	☐ 1 ☐ 2 ☐ 3 ☐ 4 ☐ 5 or ☐ ?	☐ 1 ☐ 2 ☐ 3 ☐ 4 ☐ 5 or ☐ ?
**b.** Medical records committee review of procedures	☐ 1 ☐ 2 ☐ 3 ☐ 4 ☐ 5 or ☐ ?	☐ 1 ☐ 2 ☐ 3 ☐ 4 ☐ 5 or ☐ ?
**c.** Other (Specify):	☐ 1 ☐ 2 ☐ 3 ☐ 4 ☐ 5 or ☐ ?	☐ 1 ☐ 2 ☐ 3 ☐ 4 ☐ 5 or ☐ ?
**B. TIDES Care Model Component** Primary care clinicians are provided with quick reference cards and clinician guides	☐ 1 ☐ 2 ☐ 3 ☐ 4 ☐ 5 or ☐ ?	☐ 1 ☐ 2 ☐ 3 ☐ 4 ☐ 5 or ☐ ?
**1. Implementation Option** Clinicians have access to depression improvement materials via :		
**a.** The TIDES web site	☐ 1 ☐ 2 ☐ 3 ☐ 4 ☐ 5 or ☐ ?	☐ 1 ☐ 2 ☐ 3 ☐ 4 ☐ 5 or ☐ ?
**b.** Hard copy	☐ 1 ☐ 2 ☐ 3 ☐ 4 ☐ 5 or ☐ ?	☐ 1 ☐ 2 ☐ 3 ☐ 4 ☐ 5 or ☐ ?
**c.** Other (Specify):	☐ 1 ☐ 2 ☐ 3 ☐ 4 ☐ 5 or ☐ ?	☐ 1 ☐ 2 ☐ 3 ☐ 4 ☐ 5 or ☐ ?

Comments related to items in this section:

XII. **Community Outreach**	**COLUMN A** **How *important* is this item to improving depression care in your practice setting?** Importance Rating Low (1) ⇦ ⇨ High (5) OR ? Check one box for each item	**COLUMN B** **How *feasible* is it to implement this item in your practice setting?** Feasibility Rating Low (1) ⇦ ⇨ High (5) OR ? Check one box for each item
**A. TIDES Care Model Component** Key stakeholders in the community are notified about the project.	☐ 1 ☐ 2 ☐ 3 ☐ 4 ☐ 5 or ☐ ?	☐ 1 ☐ 2 ☐ 3 ☐ 4 ☐ 5 or ☐ ?
**1. Implementation Option** Notifying these key stakeholders:		
**a.** Vet Centers	☐ 1 ☐ 2 ☐ 3 ☐ 4 ☐ 5 or ☐ ?	☐ 1 ☐ 2 ☐ 3 ☐ 4 ☐ 5 or ☐ ?
**b.** Contract care providers	☐ 1 ☐ 2 ☐ 3 ☐ 4 ☐ 5 or ☐ ?	☐ 1 ☐ 2 ☐ 3 ☐ 4 ☐ 5 or ☐ ?
**c.** Other (Specify):	☐ 1 ☐ 2 ☐ 3 ☐ 4 ☐ 5 or ☐ ?	☐ 1 ☐ 2 ☐ 3 ☐ 4 ☐ 5 or ☐ ?
**d.** Other (Specify):	☐ 1 ☐ 2 ☐ 3 ☐ 4 ☐ 5 or ☐ ?	☐ 1 ☐ 2 ☐ 3 ☐ 4 ☐ 5 or ☐ ?

Comments related to items in this section:
