# Supplementary material for: A social marketing approach to implementing evidence-based practice in VHA QUERI: the TIDES depression collaborative care model
Source: Implement Sci. 2009 Sep 28;4:64. doi: 10.1186/1748-5908-4-64 (PMC2762953; doi:10.1186/1748-5908-4-64)
Supplement: Additional file 2 — TIDES Care Manager Functional Statement. Summary of responsibilities and competencies for depression care managers. [file 1748-5908-4-64-S2.PDF]

FUNCTIONAL STATEMENT: TIDES DEPRESSION CARE MANAGERS

The VISN 23 Depression Care Manager functions as a case manager, consultant, and educator while providing collaborative care to outpatients in Primary Care at _____ (site). The assessment and collaborative care provided by this professional nurse is performed via telephone contact with referred veterans, via consult request from their primary care provider. The population served is primarily adult and older adult males, with a mean age range of 55-75 years, and includes veteran outpatients with chronic medical conditions who have screened positive for depression symptoms.

I. The primary responsibilities of the Depression Care Manager (DCM) include the following activities:

- Uses the nursing process or the analytical framework of an accepted nursing theory for the delivery of patient management.
- Provides nursing clinical leadership with tools to improve and sustain the quality and effectiveness of depression care.
- Assists medical centers in meeting the OQP Depression Performance Measure.
- Assists with coordination of the TIDES depression initiatives for ____ (site).
- Forecasts new knowledge needs for providers, veterans, and staff in the promotion and advancement of depression care.
- Collaborates with other disciplines in evaluating research activities and programs.
- Disseminates TIDES information to site PC/SM and MH Service Lines and to the wider nursing audience within ____ (site).
- Responds to referrals from PCPs in a timely manner.
- Provides patient education in areas of depression management/understanding, health promotion and functional restoration.
- Provides follow-up of patient progress and treatment adherence with reassessments and communication with their mental health and primary care providers.
- Assists in the development and implementation of patient treatment plans.

- Obtains a health history of referred patients: data collection includes lifestyle patterns; pain issues; sleep/activity levels; support systems; cultural, social and ethnic factors; patient treatment preferences and goals.
- Conducts an assessment of depression symptoms using a validated assessment tool.
- Consults TIDES collaborating psychiatrists on patient management issues.
- Actively participates with TIDES Collaborative Committee; DCM weekly conference calls.

II. Key competencies required for this position include:

- Knowledge of current treatment for depression, the key symptoms of depression and patient manifestations.
- Knowledge of assessment and case management of patients.
- Knowledge of medications prescribed for patients with depression, including patient responses and possible adverse reactions.
- Ability to use VA computer system and VistA-based clinical informatics.
- Awareness of key contacts at the participating medical centers and in the patient's community in order that timely referrals/consultations can be generated.
- Knowledge of the roles and responsibilities of researchers in conducting research activities with human participants.
- Ability to communicate effectively, both orally and in writing, with professional, administrative, technical staff and patients.
