# Supplementary material for: A social marketing approach to implementing evidence-based practice in VHA QUERI: the TIDES depression collaborative care model
Source: Implement Sci. 2009 Sep 28;4:64. doi: 10.1186/1748-5908-4-64 (PMC2762953; doi:10.1186/1748-5908-4-64)
Supplement: Additional file 3 — Selected Screen Shots from Initial Assessment Dialog. Computerized tool used by depression care managers for initial assessment of referred veterans. [file 1748-5908-4-64-S3.PDF]

DEPRESSION/TIDES INITIAL ASSESSMENT

Begin assessment

( ) Declined/unable to do assessment

(•) Depression Assessment (start with PHQ-9)

PHQ-9

(•) Record PHQ-9 score: <<<PHQ-9 SCORE (TIDES)>>>

PHQ-9 score:

* ______

PHQ-9 = 0 or PHQ-9 > 0

( ) PHQ-9 = 0

(•) PHQ-9 > 0

Initial symptom difficulty

( ) Not difficult

( ) Somewhat difficult

(•) Very difficult

( ) Extremely difficult

ASSESSMENT SUMMARY AND EVIDENCE-BASED RECOMMENDATIONS:

Probable depression DSM:

( ) 1. Transient low mood; does not have depression symptoms at this time (PHQ-9 is less than 5). Not currently appropriate for care management, but re-consult if symptoms worsen.

( ) 2. Symptoms of depression (PHQ-9 is 5-9) but not MDD; mo prior history, no dysthymia. Likely to respond to Primary Care interventions such as stress reduction, social support, exercise. Depression Care Manager will provide education & watchful waiting & recheck at 3 months.

( ) 3. Bereavement, lost a spouse, close relative in the past two months. Major depression treatment not appropriate. Care manager will provide education and watchful waiting with recheck at 2 months.

( ) 4. Dysthymia with feelings of depression and impaired functioning over last two years, and PHQ-9 is between 4 and 9. Treat with antidepressant or psychotherapy and care management.

( ) 5. Probable major depression (PHQ-9 greater than 9) or incipient relapse (PHQ-9 is 5-9 with history of depression). Patient prefers watchful waiting.

( ) 6. Probable major depression (PHQ-9 greater than 9) or incipient relapse (PHQ-9 is 5-9 with history of depression). Treat with antidepressants or psychotherapy and care management.

(•) 7. Dysthymia plus major depression; feelings of depression and impaired functioning over last two years and PHQ-9 greater than 9. Treat with antidepressants or psychotherapy and care management.

Care plan suggestions:

- [x] Watchful waiting
- [x] Consider antidepressant
- [x] Continue antidepressant
- [ ] Consider referral to Mental Health
- [ ] Consider adjusting dose or changing medication
- [ ] Consider treatment after further evaluation/treatment of:
- [ ] Consider lab testing on next primary care visit for:
- [ ] Patient needs follow-up with Primary Care Provider for medication management in (number of weeks):
- [ ] Other
- [ ] Comments on care plan suggestions:

Concomitant conditions and recommendations:

- [ ] None
- [x] ETOH with depression; refer to Mental Health/Substance abuse treatment.
- [ ] ETOH with depression. ETOH below recommended limits.

- [ ] ETOH, no depression; refer to Mental Health/Substance abuse treatment.
- [ ] ETOH, no depression. ETOH below recommended limits.
- [ ] Frequent anxiety or panic with depression; medications & frequent follow-up.
- [ ] Frequent anxiety or panic attacks without depression; refer to Mental Health.
- [ ] Substance use other than ETOH; refer to Mental Health/Substance Abuse treatment.
- [ ] Bipolar disorder; refer to Mental Health.
- [ ] Psychosis; urgent referral to Mental Health.
- [ ] PTSD screen positive; refer to Mental Health/PTSD treatment.
- [ ] Depressive response to non-depressive medication.
- [ ] Recent returning combat veteran (OEF/OIF); refer to specialty program.
- [ ] Significant pain; evaluation/treatment of pain may improve mood.
- [ ] Low social support, isolation; patient should benefit from longer treatment, frequent follow-up.
- [ ] Multiple physical symptoms; patient should benefit from longer treatment, frequent follow-up.
- [ ] Severe sleep disorder symptoms; refer for assessment.
- [ ] Severe loss of appetite and weight loss; refer for nutritional evaluation.
- [ ] Patient is smoking; refer for smoking cessation.
- [ ] Poor diabetic control; refer for diabetic education.
- [ ] Non-prescription medications of possible concern; refer for Primary Care evaluation.
- [ ] Needs activation; encourage interests, hobbies, pleasurable activities.

Patient treatment preferences:

- [ ] Medications
- [ ] Psychotherapy
- [ ] Medication AND psychotherapy
- [ ] Self-help and monitoring
- [ ] Other

Patient questions/concerns:

- (•) Yes
- ( ) None at this time

- [ ] Indications for using antidepressants:
- [ ] Indications for referring to psychotherapy:
- [ ] Mental Health specialty referral:

PROPOSED CARE MANAGER PLAN:

- ( ) Care Manager will follow patient for up to six months for depressive symptoms and treatment compliance unless otherwise directed by Primary Care or Mental Health.
- ( ) Care Manager will follow patient for up to six months for alcohol misuse unless otherwise directed by Primary Care or Mental Health
- ( ) Unless otherwise directed, Care Manager will refer patient to Mental Health.
- [ ] Patient is currently being followed by Mental Health and Care Manager will monitor for adherence to appointments.
- [ ] Patient does not meet protocol for care management. Please contact the undersigned nurse care manager.
- [ ] Patient will be discussed with supervising psychiatrist.
- [ ] Other

- [ ] Comments on care plan suggestions:
- [ ] Comments on assessment:

FULL ASSESSMENT DETAILS:

DEPRESSION SYMPTOM SCREENING:

Depressive symptoms in the last 2 weeks:

- [ ] Little interest or pleasure
- [x] Feeling down, depressed, or hopeless

- ( ) Trouble falling or staying asleep
- ( ) Sleeping too much
- [ ] Feeling tired or having little energy
- ( ) Poor appetite
- ( ) Overeating
- [ ] Feeling bad about yourself
- [x] Trouble concentrating on things
- (•) Moving or speaking slowly
- ( ) Being fidgety or restless
- [ ] Thoughts that you would be better off dead or of hurting yourself in some way

Number of symptoms: (enter value 0-9)

* [ ]

MDD trigger symptoms:

- ( ) Yes
- ( ) No

- [ ] Comments on depression screening:

- [x] RISK ASSESSMENT AND MANAGEMENT: TIDES Suicide Algorithm

Suicidal ideation/ Homicidal ideation

[Ask suicide questions if medical center directive requires it or if patient expressed current thoughts of suicide on PHQ-9 or if suicidal ideation is noted at other points in the assessment. Even in the absence of a plan, discuss all patients with any risk factors and suicidal ideation with Mental Health specialist with the week.]

<<<SUICIDE RISK (TIDES)>>>

Suicidal/Homicidal

- [x] Suicidal ideation:

Select one (suicidal ideation)

- ( ) Verbalizes, wouldn't consider suicide/harm to self
- ( ) Has passive thoughts of death
- ( ) Has thoughts about act of suicide but no plan
- ( ) Has suicide plan but says he/she won't act on it
- (•) Has suicide plan and may act on it (INVOKE MEDICAL CENTER SUICIDE PROTOCOL) AND close consult.

```
*************************************************************
*                                                           *
*  High Risk for Suicide - Immediate Intervention Required  *
*                                                           *
*************************************************************
```

VA PSHCS Telephone Nurse Care Manager Suicide Prevention Protocol:

- [ ] 1. Write down the number where patient is calling from. Obtain additional contact numbers for patient and exact physical location (address) if possible.
- [ ] 2. Assess for severity (intent, plan, previous attempts, current substance use/abuse, access to weapons or other means).
- [ ] 3. Assess for safety (support system; has he/she told anybody else?; ability to come in to be seen, go to Emergency Department, or contact Mental Health if condition worsens; ability to follow up with current treatment plan; any currently restricted access to means).
- [ ] 4. If you are concerned about immediate safety, ask if he/she is currently alone, and are minors present. If not, ask to speak to another person (obtain additional assessment information and develop a safety plan).
- [ ] 5. Before hanging up, insure patient has the VA's 24hr hotline number: 1-800-273-TALK (1-800-273-8255) and possibly another emergency number.
- [ ] 6. Contact 911 for safety check if needed (or have a co-worker call while you stay engaged with the patient). Obtain police information (who responded? What actions were taken?).
- [ ] 7. Notify immediate supervisor or designated surrogate.
- [ ] 8. Notify Suicide Prevention Coordinator.

- [ ] Other risk factors for suicide:

Suicidal ideation management:

- [ ] Patient's case will be discussed with Mental Health specialist.

- [ ] Mental Health appointment is being made.
- [ ] Suicidal ideation will continue to be monitored.
- [ ] Care Manager discussed with patient not to harm self, assessed and reinforced social support, persons to contact, and what patient has done in the past to feel better or resist suicide.

- [x] Homicidal ideation:

(•) Yes, threatened; homicide protocol initiated

VA Puget Sound Telephone Nurse Care Manager Homicide Prevention Protocol:

- [ ] 1. Write down the number where the patient is calling from. Obtain additional contact numbers for patient and exact physical location (address) if possible.
- [ ] 2. Assess for severity (intent, plan, previous attempts, current substance use/abuse, access to weapons or other means).
- [ ] 3. Assess for safety (support system; has he/she told anybody else?; ability to come in to be seen, go to Emergency Department, or contact Mental Health if condition worsens; ability to follow up with current treatment plan; any currently restricted access to means).
- [ ] 4. If you are concerned about immediate safety, ask if he/she is currently alone. If not, ask to speak to another person (obtain additional assessment information and develop a safety plan).
- [ ] 5. Ask the name, phone number, address and current location of intended victim.
- [ ] 6. Contact 911 for safety check if needed (or have a co-worker call while you stay engaged with the patient.)
- [ ] 7. Contact immediate supervisor (Dr. Felker pager 570-2782) or designated surrogate.

( ) Yes, threatened; homicide policy not initiated

( ) No

- [ ] Comments on risk assessment and management:

DEPRESSION HISTORY:

Depression diagnosed in past?

(•) Yes

( ) No

( ) Not sure

Prior treatment for depression?

(•) Yes, previous antidepressants and psychotherapy

With antidepressants

( ) 1 time

( ) 2 times

( ) 3 times

( ) 4+ times

With psychotherapy

( ) 1 group session

( ) Multiple group sessions

( ) 1-2 individual sessions

( ) Multiple individual sessions

( ) Yes, with antidepressants

( ) Yes, with psychotherapy

( ) No, never treated

Dysthymia symptoms?
[In the past 2 years, have you felt depressed or sad most days, even if you felt okay sometimes?]

( ) Yes

( ) No

Depressive symptoms last 2 years:

- [ ] Little interest or pleasure
- [ ] Feeling down, depressed, or hopeless

( ) Trouble falling or staying asleep

- ( ) Sleeping too much
- [ ] Feeling tired or having little energy
- ( ) Poor appetite
- ( ) Overeating
- [ ] Feeling bad about yourself
- [ ] Trouble concentrating on things
- ( ) Moving or speaking slowly
- ( ) Being fidgety or restless
- [ ] Thoughts that you would be better off dead or of hurting yourself in some way

Number of dysthymia symptoms: (enter value 0-9)

* ______

Functioning over the last 2 years:

- (•) Impaired function MORE than half the time
- ( ) Impaired function LESS than half the time

- [ ] Comments on depression history: (include medication names if relevant)

ANTIDEPRESSANT MEDICATION COMPLIANCE AND SIDE EFFECTS:

Currently on antidepressants?

- (•) Yes - less than two months

Taking antidepressants as directed?

- ( ) Yes
- ( ) No

Last Rx fill on time?

- ( ) Yes
- ( ) No

Antidepressant medication side effects?

- (•) Yes
  - ( ) Activation (over-stimulation) -
  - ( ) Agitation -
  - ( ) Anxiety -
  - ( ) Blurred vision -
  - ( ) Confusion -
  - ( ) Constipation -
  - ( ) Diarrhea -
  - ( ) Difficulty urinating -
  - ( ) Dizziness/light-headedness -
  - ( ) Dry mouth -
  - ( ) Headaches -
  - ( ) Irritability/anger -
  - ( ) Loss of appetite -
  - ( ) Memory problems -
  - ( ) Muscle weakness -
  - ( ) Nausea/upset stomach -
  - ( ) Nightmares -
  - ( ) Sedation -
  - ( ) Sexual dysfunction -
  - ( ) Sweating -
  - ( ) Tachycardia -
  - ( ) Tremor -
  - ( ) Other -
- ( ) No

- ○ Yes - greater than two months
- ○ No, but ordered
- ○ No
- ○ Comments on medication compliance and/or side effects:

SYMPTOMS:

Describe sleep:

- ○ Not problematic
- ○ Problematic

Number of hours sleep: (enter number)

* [ ]

Describe appetite:

- ○ Adequate
- ○ Poor
- ○ Overeating
- ○ Inconsistent eating pattern
- ○ Elderly, not eating
- ○ Elderly, not eating, referral initiated

Weight change:

- ○ No change
- ○ Recent gain
- ○ Recent loss

Symptoms in last month:

- ☐ None
- ☐ Activation
- ☐ Agitation
- ☐ Anxiety
- ☐ Blurred vision
- ☐ Confusion
- ☐ Constipation
- ☐ Diarrhea
- ☐ Difficulty urinating
- ☐ Dizziness/light-headedness
- ☐ Dry mouth
- ☐ Headaches
- ☐ Irritability/anger
- ☐ Memory problems
- ☐ Muscle weakness
- ☐ Nausea/upset stomach
- ☐ Nightmares
- ☐ Sedation
- ☐ Sexual dysfunction
- ☐ Sweating
- ☐ Tachycardia
- ☐ Tremor
- ☐ Other

CO-MORBIDITIES:

Medical co-morbidities:

- ☐ [illegible]

- ☐ None
- ☐ Active smoker
- ☐ Cancer
- ☐ Hx of MI, CAD, CHF
- ☐ Hx of lung disease, emphysema, asthma, bronchitis
- ☐ Pain
- ☐ Other

Impact on physical health:

- ○ Not at all
- ○ Somewhat
- ○ Quite a bit
- ○ Significant

New medications past 2 months

- ☐ None
- ☐ HTN/cardio
- ☐ Sedative/hypnotic agents
- ☐ Anti-inflammatory agents
- ☐ Analgesics
- ☐ Hormones
- ☐ Parkinson's agents
- ☐ Anti-ulcer
- ☐ Antineoplastic agents
- ☐ Stimulants

☐ Comments on medical co-morbidities:

<<<NON-VA MEDS (PAST 2 MOS)>>>

Other medications, herbal supplements, drugs:

- ◉ Yes (Non-VA meds or other illicit drugs)
  - Non-VA meds (both modes)
    - ○ Herbal supplements/OTC meds: (goes to progress note only)
  - ☐ Other (illicit):
- ○ No

Mental Health Co-morbidities:

ETOH: History of abuse?

- ○ Yes
- ○ No

ETOH in past year?

- ◉ Yes

ETOH: Currently drinking?

- ○ No - not currently drinking
- ◉ (MALE) - Yes currently drinking

ETOH: Current # of drinks/week (Male):

- ○ 1 to 3 drinks per week
- ○ 4 to 6 drinks per week
- ○ 7 to 10 drinks per week
- ○ 11 to 14 drinks per week
- ○ More than 14 drinks per week

ETOH: Current number of drinks per occasion (Male):

- ○ 1 drink per occasion
- ○ 2 drinks per occasion
- ○ 3 drinks per occasion

- More than 4 drinks per occasion

ETOH: Assessment of use (Male) - Less than 14 drinks per week AND maximum 4 drinks per occasion?

- Yes
- No

- (FEMALE) - Yes currently drinking

ETOH: Brief intervention required?

- (selected) Yes
- No

- No

Does patient want treatment for ETOH?

- Yes
- No

ETOH in CPRS active problem list?

- Yes
- No

Drug Abuse: Does patient report significant abuse?

- Yes, wants treatment
- Yes, does NOT want treatment
- No

Drug abuse in CPRS active problem list?

- Yes
- No

Anxiety/Panic: Has patient felt anxious/frightened/panicked in the past month?

- Yes, MORE than 1/2 the days in the past month
- Yes, LESS than 1/2 the days in the past month
- Denies anxiety/panic

Anxiety/Panic: Does patient want referral?

- Wants referral
- Doesn't want referral

Anxiety/panic in CPRS active problem list?

- Yes
- No

PTSD: Diagnosed with PTSD?

- Yes
- No
- Not sure

PTSD in CPRS active problem list?

- Yes
- No

PTSD factors from VISTA (last 2 reminders done): <<<PTSD FACTORS (TIDES)>>>

PTSD FACTORS:

Nightmares:

- Yes
- No

Avoidance:

- Yes
- No

On Guard:

- ( ) Yes
- ( ) No

Detachment:

- ( ) Yes
- ( ) No

PTSD Referral:

- ( ) Reports 2 or more significant PTSD factors and WANTS referral
- ( ) Reports 2 or more significant PTSD factors and does NOT WANT referral
- ( ) Already in treatment for PTSD
- ( ) 0-1 factors and not in treatment for PTSD

Bipolar:

Bipolar: Diagnosed bipolar?

- ( ) Yes
- ( ) No
- ( ) Not sure

Bipolar in CPRS active problem list?

- ( ) Yes
- ( ) No

[ ] BIPOLAR SCREENING: (select this item to perform the Bipolar Screen)

Hallucinations/delusions?

- (•) Yes
- ( ) No
- ( ) Not sure

[ ] Comments on mental health co-morbidities:

FUNCTIONING, SUPPORT, AND STRESSORS:

Employment status:

- [ ] Working full time
- [ ] Working part time
- [ ] Medical disability
- [ ] Unemployed, seeking employment
- [ ] Unemployed, not seeking employment
- [ ] Retired
- [ ] Other

Volunteers:

- ( ) Yes
- ( ) No

Hobbies/activities:

- ( ) Yes
- ( ) No

Marital status:

- ( ) Married
- ( ) Significant other
- ( ) Separated
- ( ) Divorced
- ( ) Widowed
- ( ) Single, never married

Living situation:

- ( ) Alone
- ( ) [illegible]

- ○ Family
- ○ Other

Able to care for self?

- ○ Yes
- ○ No, dependent on others
- ○ Other

Children:

- ○ Yes (dependent children)
- ○ No children

Social/Family support:

- ○ Little/none
- ○ Adequate
- ○ Strong

Support system:

- ☐ Family
- ☐ Friends
- ☐ Church
- ☐ Social group
- ☐ Agency
- ☐ Other
- ☐ No support from family, friends, church or social group

Specific support persons?

- ○ Yes
- ○ No

Patient stressors:

- ☐ Financial
- ☐ Home
- ☐ Work
- ☐ Family
- ☐ Education
- ☐ Legal
- ☐ Own health
- ☐ Family member's health
- ☐ Loss of family/friend
- ☐ None
- ☐ Other

Service era: <<<MILITARY SERVICE - local PATIENT SERVICE in live>>>

- ○ WWII
- ○ Korea
- ○ Vietnam
- ○ 1st Gulf War
- ○ OEF/OIF
- ○ Peacetime

☐ Comments re: Functioning/Support System/Stressors:

PATIENT SELF-CARE MANAGEMENT AND EDUCATION:

☐ Previously sent material:

Self-help plan in place?

- ○ Yes
- ○ No

Doing self-help activities?

- ◯ Yes
- ◯ No

☐ Information offered/encouraged:

Information to be mailed to patient:

"Are You Feeling...Tired, Sad, Angry, Irritable, Hopeless?"
"The Cycle of Depression"
"Persons Considering Psychological Counseling Treatment for Depression"
"Frequently Asked Questions About Antidepressant Medications"
"Depression Self-Care Action Plan"
"Plan to Monitor and Prevent Future Depression"
"Suicide Prevention"

- ☐ PHQ-9 score
- ☐ PHQ-9 graph
- ☐ Contact card
- ☐ Medication instructions
- ☐ Sleep hygiene
- ☐ Diet
- ☐ Exercise
- ☐ General depression
- ☐ Self-help material
- ☐ Community resources
- ☐ Other

Potential barriers to learning:

- ☐ None
- ☐ Patient disagrees he/she may be depressed.
- ☐ Patient doesn't understand seriousness of the need for treatment.
- ☐ Patient doesn't think treatment will help.
- ☐ Patient is uncomfortable talking about personal things.
- ☐ Patient is embarrassed.
- ☐ Patient has conflicting religious beliefs.
- ☐ Patient thinks other problems are more important.
- ☐ Patient thinks treatment will take too long or be too expensive.
- ☐ Unable to write
- ☐ Homeless
- ☐ Visually impaired
- ☐ Hearing impaired

Preferred learning methods:

- ☐ Reading
- ☐ Discussion
- ☐ Lecture/class
- ☐ Demonstration
- ☐ Media (Audio-Visual)
- ☐ Computer literate

☐ Comments regarding patient education/self-management:

LOGISTICS:

- ☐ Best time to call:
- ☐ Best days to call:
- ☐ Best numbers to call:
- ☐ Patient refuses further calls:

Provider feedback

Patient's <<<PRIMARY CARE PROVIDER>>>

Designate patient's Primary Care Provider or the referring clinician as an additional signer on this note.

- [ ] List the patient's PCP <AND> any others that are additional signers on your note:

Call history: (attempts to reach the patient for THIS assessment)

- ( ) 1 try
- ( ) 2 tries
- ( ) 3 tries
- ( ) 4 tries
- ( ) 5 tries

- [ ] Encounter codes for this visit
- [ ] Additional comments:
