# Supplementary material for: A social marketing approach to implementing evidence-based practice in VHA QUERI: the TIDES depression collaborative care model
Source: Implement Sci. 2009 Sep 28;4:64. doi: 10.1186/1748-5908-4-64 (PMC2762953; doi:10.1186/1748-5908-4-64)
Supplement: Additional file 4 — Helping VA Primary Care Providers Manage Patients with Depression. Summary of TIDES collaborative depression care model from perspective of referring primary care clinician. [file 1748-5908-4-64-S4.PDF]

Helping VA Primary Care Providers
Manage Patients with Depression

What is TIDES?

In the VA, the majority of depressed patients are identified within primary care rather than mental health. TIDES (Translating Initiatives for Depression into Effective Solutions) helps you, as a primary care clinician, deliver a high-quality level of depression care without adding to your workload. You can still refer severe cases to mental health specialists.

Whom is TIDES for?

TIDES facilitates care for depressed veterans within the primary care setting.

How does TIDES work?

Every VA clinic participating in TIDES has a Depression Care Manager (DCM) who can help you manage depressed veterans. When you diagnose depression, you may send a consult to your clinic's DCM. The DCM will then follow up with the patient.

How well does TIDES work?

The program has been successfully implemented for three years across three VISNs. Patients followed for six months showed significant improvement in depression scores and overall functioning.

What does the DCM do?

The DCM calls patients, assesses their level of depression and decides what support the patients need based on specific protocols. Although DCMs do not prescribe medications, they monitor treatment progress and compliance. Primary care providers at other VA facilities who have already used this program identify *additional patient assessment* as the most valuable service the DCM performs. Other DCM activities may include patient education, patient activation, symptom monitoring and, in select cases, coordination of care between mental health and primary care. The DCM provides follow up for six months, usually by telephone. If appropriate, you may refer the patient for an additional six-month period of care management. At present, DCM activities count towards the three visits mandated by VA Depression Performance Measuring.

Who are the DCMs?

Depression Care Managers are RNs with formal training in care management and the management of depression in primary care patients. They receive supervision from psychiatrists and, typically, are centrally located with the VISN or at a VAMC. DCMs enter notes into CPRS and send them to you as a co-signer. This process helps you stay informed about the progress of your patient.

When is it inappropriate to refer to TIDES?

You should refer patients who have severe psychiatric comorbidity—severe PTSD, bipolar disorder, etc.—to mental health specialists. You should also refer patients with severe depression to these specialists. If you have questions about the appropriateness of a referral, please contact your DCM.

How do I refer a patient?

You can refer a patient by using CPRS to send a consult to the DCM.

Who is the Depression Care Manager for our VISN?

The Depression Care Manager in [Location] is [Name]. See below for contact information.

TIDES Feedback:

"Under normal circumstances, a provider sees the patient today and not again for three months. There's no contact in between to check on the patient. But with TIDES, you know the DCM is checking on patients, and it makes you feel more comfortable assessing and managing patients with depression." **—Physician**

"We've had several patients who've said, 'It's the best thing that's happened to me.' Patients can open up and talk about things they've been carrying around for a long time. They feel good being able to do that." **—Physician**

"I have heard good reports from patients who can get more information and follow up than we otherwise could provide." **—Physician**

"I told the DCM that I had emotional problems and I'm glad she called because I really thought I needed help. I didn't think so before, but I do now. Before, I would never have been comfortable talking, but I realized I needed some help, and it was comforting to know that somebody cared. On the phone, I could explain something without looking at somebody who might have thought less of me." **—Consumer**

MENTAL HEALTH QUERI
Quality Enhancement Research Initiative

TIDES is part of Mental Health QUERI, a national VA HSR&D-funded initiative to improve the quality of care and health outcomes of veterans with major depressive disorder and schizophrenia.

[Location] Depression Care Manager:

[Name]

Phone: [Number]

E-mail: [E-mail Address]
