# Supplementary material for: A social marketing approach to implementing evidence-based practice in VHA QUERI: the TIDES depression collaborative care model
Source: Implement Sci. 2009 Sep 28;4:64. doi: 10.1186/1748-5908-4-64 (PMC2762953; doi:10.1186/1748-5908-4-64)
Supplement: Additional file 5 — Understanding the TIDES Program. Two-page overview for marketing purposes that succinctly describes what TIDES is, what it accomplishes, how it works, and the evidence supporting it. [file 1748-5908-4-64-S5.PDF]

Understanding the TIDES Program

Upon request, we would be happy to supply additional information and supporting references for TIDES. For additional information, please contact:

Richard R. Owen, MD
Research Coordinator
Mental Health QUERI
(501) 257-1710
Richard.Owen2@va.gov

What is TIDES?

TIDES (**T**ranslating **I**nitiatives for **D**epression into **E**ffective **S**olutions) is an evidence-based depression collaborative care model involving both primary care and mental health specialists. It has promoted improvements in treatment adherence and recovery for veterans with depression in three VISNs (VISNs 10, 16, and 23).

TIDES implementation is being accomplished through innovative partnerships among VA clinical leaders, Mental Health QUERI and researchers in Los Angeles (Center for the Study of Healthcare Provider Behavior), Seattle (VA Puget Sound), and Little Rock (Center for Mental Healthcare and Outcomes Research).

- Patient adherence to treatment is greater than 80% across the first 1,000 patients receiving TIDES care.
- Recovery at six months is 70% among primary care patients and 50% among those referred to specialty care.
- TIDES has now been expanded to new facilities and clinics in those VISNs, and also to a fourth VISN (VISN 22).

Effective care models

Effective care models for depression, such as TIDES, involve ***collaboration*** between mental health and primary care specialists with support for:

- assessment and triage
- patient education and activation
- pro-active follow-up of primary care patients with depressive symptoms

Most effective care models, including TIDES, depend upon registered nurses who receive special training to provide this support:

- TIDES nurses serving in this capacity are called Depression Care Managers (DCMs).
- DCMs can provide this support to about 300 patients per year; generally one DCM per 10,000 PC patient population is required to provide these services.

Why is TIDES needed?

- Depression is under-diagnosed and undertreated in VA primary care in spite of its high prevalence, the well-documented adverse effects on health status and cost, and the widespread availability of effective treatments.
- Performance measures have a target adherence level of 20% for minimally adequate follow-up for a new diagnosis of depression. ***Typical VA facilities are currently at 10% or less.***
- The situation is actually worse than it appears. Many patients qualifying for this performance measure are based in mental health clinics. ***Minimally appropriate follow-up among primary care-based patients is often only around 1%.***

What does TIDES accomplish?

For veterans with Major Depressive Disorder (MDD):

- Educates and empowers patients to self-monitor, self-manage, and engage actively in their care in partnership with their providers.
- Improves safety through prompt symptom detection and management, appropriate use and monitoring of antidepressants, relapse prevention, and appropriate use of MH specialty referral.
- Improves quality of life and functioning at work and at home.

For VA Mental Health (MH) and Primary Care (PC) services:

- Increases access to appropriate MH specialty care by decreasing inappropriate referrals, no-shows, and single visits without appropriate follow-up.
- Decreases PC visits for depression-related somatic symptoms.
- Increases appropriate, effective, and cost-sensitive use of depression treatments.
- Increases compliance with performance measures.

Understanding the TIDES Program *(continued)*

How does TIDES work?

◆ PC providers refer patients to DCMs for telephone evaluation and management of depression symptoms.

◆ Utilizing protocol-based assessment tools as well as patient education and activation strategies, DCMs:

- Identify patients who meet criteria for MDD and make treatment suggestions taking into account patient preferences.
- Suggest appropriate alternative treatment for patients who do not meet MDD criteria.

◆ DCMs implement the primary care clinician's care plan for MDD.

- Plans may include antidepressants in primary care, watchful waiting, or referral to a mental health specialist (MHS).
- For patients managed within primary care, the DCM conducts brief, 10-15 minute telephone calls over six months to assess symptoms, treatment adherence, and problems with treatment to prevent relapse, and to reinforce depression knowledge and skills.
- If necessary, providers can re-refer patients to DCM for additional follow-up.

◆ Enhancements to CPRS developed as part of TIDES—including consult, note template, clinical reminder, assessment package, and administrative functions—help DCMs manage their patient panels, facilitate communication among collaborating care providers, support patient self-management, and enable clinics to monitor their improvements in depression care.

◆ The graphic illustrates the lines of communication and interaction among PC providers, DCMs, and MHSs in TIDES collaborative care.

TIDES Collaborative Care for Depression

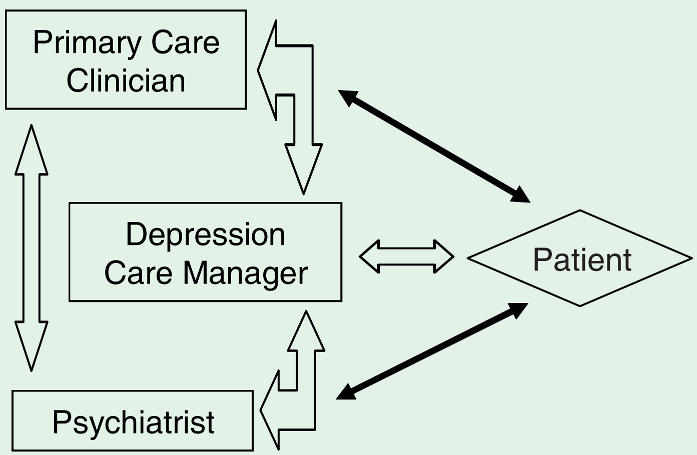

What is the target population for TIDES?

The target population is patients newly identified in primary care with symptoms of depression, who do not have symptoms indicating a psychiatric emergency, and who have a telephone.

Patients outside the target population also benefit from enhanced access to mental health services, greater depression awareness in primary care, and policy and educational developments to improve depression care.

What does the literature supporting TIDES say?

◆ Primary care assessment and initial treatment are the strongest predictors of outcomes. If care does not begin in primary care, specialty care is unlikely to occur either.

◆ In randomized, controlled trials, the type of care used in TIDES improves quality of care and outcomes for men and women of all ages, from diverse ethnic backgrounds, and in a broad range of rural and urban practice settings, including VA and managed care settings.

◆ Outcomes remain improved for two to five years—including improvements in treatment adherence, recovery, social and role functioning, financial status, and patient satisfaction.

◆ Outcomes are equivalent for in-person and telephone care management. Depression symptoms, including suicidality risk, can be safely and effectively assessed by telephone in TIDES care.

◆ Cost-effectiveness studies in a variety of settings, ***including the VA***, show low cost per effect.

Mental Health QUERI is a national VA HSR&D-funded initiative to improve the quality of care and health outcomes of veterans with major depressive disorder and schizophrenia.
